# Global estimates on the number of people blind or visually impaired by age-related macular degeneration: a meta-analysis from 2000 to 2020

**DOI:** 10.1038/s41433-024-03050-z

**Published:** 2024-07-04

**Authors:** Vision Loss Expert Group of the Global Burden of Disease Study, João M. Furtado, Jost B. Jonas, Ian Tapply, Arthur G. Fernandes, Maria Vittoria Cicinelli, Alessandro Arrigo, Nicolas Leveziel, Serge Resnikoff, Hugh R. Taylor, Tabassom Sedighi, Seth Flaxman, Maurizio Battaglia Parodi, Mukkharram M. Bikbov, Tasanee Braithwaite, Alain Bron, Ching-Yu Cheng, Nathan Congdon, Monte A. Del Monte, Joshua R. Ehrlich, Tim Fricke, David Friedman, Gus Gazzard, M. Elizabeth Hartnett, Rim Kahloun, John H. Kempen, Moncef Khairallah, Rohit C. Khanna, Judy E. Kim, Van Charles Lansingh, Janet Leasher, Kovin S. Naidoo, Vinay Nangia, Michal Nowak, Konrad Pesudovs, Tunde Peto, Pradeep Ramulu, Fotis Topouzis, Mitiadis Tsilimbaris, Ya Xing Wang, Ningli Wang, Rupert R. A. Bourne, the GBD 2019 Blindness and Vision Impairment Collaborators, João M. Furtado, Jost B. Jonas, Arthur G. Fernandes, Maria Vittoria Cicinelli, Nicolas Leveziel, Paul Svitil Briant, Theo Vos, Serge Resnikoff, Florian Fischer, Yohannes Habtegiorgis Abate, Mohammad Abdollahi, Tadele Girum Girum Adal, Isaac Yeboah Addo, Kishor Adhikari, Prerna Agarwal, Antonella Agodi, Williams Agyemang-Duah, Aqeel Ahmad, Hamid Ahmadieh, Hooman Ahmadzadeh, Fares Alahdab, Ahmad Samir Alfaar, Robert Kaba Alhassan, Syed Shujait Shujait Ali, Louay Almidani, Sofia Androudi, Abhishek Anil, Anayochukwu Edward Anyasodor, Jalal Arabloo, Mubarek Yesse Ashemo, Seyyed Shamsadin Athari, Desta Debalkie Atnafu, Alok Atreya, Melese Kitu Ayalew, Yared Asmare Aynalem, Zewdu Bishaw Aynalem, Ahmed Y. Azzam, Sara Bagherieh, Ruhai Bai, Martina Barchitta, Mainak Bardhan, Till Winfried Bärnighausen, Nebiyou Simegnew Bayileyegn, Fatemeh Bazvand, Ahmet Begde, Babak Behnam, Akshaya Srikanth Bhagavathula, Sonu Bhaskar, Gurjit Kaur Bhatti, Jasvinder Singh Bhatti, Bagas Suryo Bintoro, Marina G. Birck, Tasanee Braithwaite, Katrin Burkart, Yasser Bustanji, Florentino L. Caetano dos Santos, Vera L. A. Carneiro, Muthia Cenderadewi, Vijay Kumar Chattu, Dinh-Toi Chu, Kaleb Coberly, Natália Cruz-Martins, Omid Dadras, Xiaochen Dai, Ana Maria Dascalu, Mohsen Dashti, Anna Dastiridou, Maedeh Dastmardi, Xinlei Deng, Nikolaos Dervenis, Vinoth Gnana Chellaiyan Devanbu, Mengistie Diress, Shirin Djalalinia, Joshua R. Ehrlich, Michael Ekholuenetale, Temitope Cyrus Ekundayo, Iman El Sayed, Muhammed Elhadi, Mehdi Emamverdi, Ambaw Abebaw Emrie, Adeniyi Francis Fagbamigbe, Ayesha Fahim, Umar Farooq, Hossein Farrokhpour, Ali Fatehizadeh, Alireza Feizkhah, Lorenzo Ferro Desideri, Getahun Fetensa, Bikila Regassa Feyisa, Seth Flaxman, Ali Forouhari, Matteo Foschi, Kayode Raphael Fowobaje, Aravind P. Gandhi, Tilaye Gebru Gebi, Miglas W. Gebregergis, Mesfin Gebrehiwot, Brhane Gebremariam, Gebreamlak Gebremedhn Gebremeskel, Yibeltal Yismaw Gela, Molalegn Mesele Gesese, Khalil Ghasemi Falavarjani, Fariba Ghassemi, Sherief Ghozy, Mahaveer Golechha, Pouya Goleij, Sapna Gupta, Veer Bala Gupta, Vivek Kumar Gupta, Teklehaimanot Gereziher Haile, Semira Goitom Hailu, Arvin Haj-Mirzaian, Aram Halimi, Shahin Hallaj, Billy Randall Hammond, Ikramul Hasan, Hamidreza Hasani, Hossein Hassanian-Moghaddam, Mahsa Heidari-Foroozan, Sung Hwi Hong, Praveen Hoogar, Mehdi Hosseinzadeh, Chengxi Hu, Hong-Han Huynh, Mustapha Immurana, Chidozie C. D. Iwu, Louis Jacob, Abdollah Jafarzadeh, Mihajlo Jakovljevic, Shubha Jayaram, Mohammad Jokar, Nitin Joseph, Charity Ehimwenma Joshua, Gebisa Guyasa Kabito, Laleh R. Kalankesh, Sagarika Kamath, Himal Kandel, Ibraheem M. Karaye, Hengameh Kasraei, Gbenga A. Kayode, Shemsu Kedir, Yousef Saleh Khader, Himanshu Khajuria, Moawiah Mohammad Khatatbeh, Mahalaqua Nazli Khatib, Zahra Khorrami, Yun Jin Kim, Adnan Kisa, Sezer Kisa, Soewarta Kosen, Ai Koyanagi, Kewal Krishan, Chandrakant Lahariya, Tri Laksono, Dharmesh Kumar Lal, Van Charles Lansingh, Trang D. T. Le, Janet L. Leasher, Munjae Lee, Seung Won Lee, Wei-Chen Lee, Stephen S. Lim, Xuefeng Liu, Alireza Mahmoudi, Razzagh Mahmoudi, Kashish Malhotra, Vahid Mansouri, Roy Rillera Marzo, Andrea Maugeri, Colm McAlinden, Tesfahun Mekene Meto, Abera M. Mersha, Tomislav Mestrovic, Ephrem Tesfaye Mihretie, Mehdi Mirzaei, Prasanna Mithra, Nouh Saad Mohamed, Soheil Mohammadi, Abdulwase Mohammed, Ali H. Mokdad, Hossein Molavi Vardanjani, Mohammad Ali Moni, Fateme Montazeri, Maryam Moradi, Parsa Mousavi, Ahmed Nuru Muhamed, Admir Mulita, Kovin S. Naidoo, Ganesh R. Naik, Shumaila Nargus, Zuhair S. Natto, Biswa Prakash Nayak, Mohammad Negaresh, Hadush Negash, Seyed Aria Nejadghaderi, Dang H. Nguyen, Hien Quang Nguyen, Phat Tuan Nguyen, Van Thanh Nguyen, Robina Khan Niazi, Mamoona Noreen, Ogochukwu Janet Nzoputam, Ismail A. Odetokun, Andrew T. Olagunju, Matthew Idowu Olatubi, Obinna E. Onwujekwe, Michal Ordak, Uchechukwu Levi Osuagwu, Nikita Otstavnov, Mayowa O. Owolabi, Jagadish Rao Padubidri, Parsa Panahi, Ashok Pandey, Shahina Pardhan, Jay Patel, Venkata Suresh Patthipati, Shrikant Pawar, Arokiasamy Perianayagam, Ionela-Roxana Petcu, Hoang Tran Pham, Ibrahim Qattea, Pankaja Raghav Raghav, Fakher Rahim, Vafa Rahimi-Movaghar, Mohammad Hifz Ur Rahman, Mosiur Rahman, Premkumar Ramasubramani, Ahmed Mustafa Rashid, Annisa Utami Rauf, Elrashdy Moustafa Mohamed Redwan, Nazila Rezaei, Priyanka Roy, Zahra Saadatian, Siamak Sabour, Basema Saddik, Umar Saeed, Sare Safi, Sher Zaman Safi, Amene Saghazadeh, Fatemeh Saheb Sharif-Askari, Narjes Saheb Sharif-Askari, Amirhossein Sahebkar, Joseph W. Sakshaug, Saina Salahi, Sarvenaz Salahi, Mohamed A. Saleh, Yoseph Leonardo Samodra, Vijaya Paul Samuel, Abdallah M. Samy, Aswini Saravanan, Monika Sawhney, Mete Saylan, Sayed Mansoor Sediqi, Siddharthan Selvaraj, Yashendra Sethi, Allen Seylani, Jaffer Shah, Samiah Shahid, Moyad Jamal Shahwan, Masood Ali Shaikh, Muhammad Aaqib Shamim, Maryam Shayan, Mika Shigematsu, Aminu Shittu, Seyed Afshin Shorofi, Emmanuel Edwar Siddig, Juan Carlos Silva, Jasvinder A. Singh, Paramdeep Singh, Eirini Skiadaresi, Raúl A. R. C. Sousa, Chandrashekhar T. Sreeramareddy, Vladimir I. Starodubov, Birhan Tsegaw Taye, Jansje Henny Vera Ticoalu, Guesh Mebrahtom Tsegay, Miltiadis K. Tsilimbaris, Saif Ullah, Muhammad Umair, Sahel Valadan Tahbaz, Nuwan Darshana Wickramasinghe, Guadie Sharew Wondimagegn, Lin Yang, Arzu Yiğit, Dong Keon Yon, Naohiro Yonemoto, Yuyi You, Mikhail Sergeevich Zastrozhin, Hanqing Zhao, Peng Zheng, Makan Ziafati, Magdalena Zielińska, Jaimie D. Steinmetz, Rupert R. A. Bourne

**Affiliations:** 1 Ribeirão Preto Medical School, University of São Paulo, Ribeirão Preto, Brazil; 2 Department of Ophthalmology, Medical Faculty Mannheim, Heidelberg University, Heidelberg, Germany; 3 Department of Ophthalmology, Cambridge University Hospitals, Cambridge, UK; 4 Federal University of Sao Paolo, Sao Paolo, SP Brazil; 5 University of Calgary, Calgary, AB Canada; 6 School of Medicine, Vita-Salute San Raffaele University, Milan, Italy; 7 Department of Ophthalmology, IRCCS San Raffaele Scientific Institute, Milan, Italy; 8 Scientific Institute San Raffaele Hospital, Vita-Salute University, Milan, Italy; 9 University of Poitiers, Poitiers, France; 10 CHU de Poitiers, Poitiers, France; 11 Brien Holden Vision Institute, Sydney, NSW Australia; 12 School of Optometry and Vision Sciences, Faculty of Medicine, University of New South Wales, Sydney, NSW Australia; 13 School of Population and Global Health, University of Melbourne, Carlton, VIC, Australia; 14 Vision and Eye Research Institute, Anglia Ruskin University, Cambridge, UK; 15 Department of Computer Science, University of Oxford, Oxford, UK; 16 Department of Ophthalmology, Vita-Salute San Raffaele University, Milano, Italy; 17 Ufa Eye Research Institute, Ufa, Russia; 18 School of Life Course and Population Sciences, King’s College London, London, UK; 19 The Medical Eye Unit, Guy’s and St Thomas’ NHS Foundation Trust, London, UK; 20 University Hospital, Dijon, France; 21 National University of Singapore, Singapore, Singapore; 22 Singapore Eye Research Institute, Singapore, Singapore; 23 Queen’s University Belfast, Northern Ireland, Belfast, UK; 24 Orbis International, New York, USA; 25 Zhongshan Ophthalmic Center, Sun Yat-sen University, Guangzhou, China; 26 University of Michigan, Ann Arbor, USA; 27 Kellogg Eye Center, Ann Arbor, USA; 28 Institute for Social Research, University of Michigan, Ann Arbor, USA; 29 Department of Ophthalmology and Visual Sciences, University of Michigan, Ann Arbor, USA; 30 Australian College of Optometry, Melbourne, Vic Australia; 31 University of Melbourne, Melbourne, Vic Australia; 32 UNSW Sydney, Sydney, NSW Australia; 33 Mass Eye and Ear, Harvard Medical School, Boston, USA; 34 Institue of Ophthalmology UCL & NIHR Biomedical Research Centre, London, UK; 35 Stanford University, Stanford, USA; 36 Associated Ophthalmologists of Monastir, Monastir, Tunisia; 37 Department of Ophthalmology, Harvard University, Boston, MA USA; 38 Eye Unit, MyungSung Medical College, Addis Ababa, Ethiopia; 39 Department of Ophthalmology, Addis Ababa University, Addis Ababa, Ethiopia; 40 Sight for Souls, Bellevue, WA USA; 41 Fattouma Bourguiba University Hospital, University of Monastir, Monastir, Tunisia; 42 Allen Foster Community Eye Health Research Centre, Gullapalli Pratibha Rao International Centre for Advancement of Rural Eye care, L.V. Prasad Eye Institute, Hyderabad, India; 43 Brien Holden Eye Research Centre, L.V. Prasad Eye Institute, Banjara Hills, Hyderabad, India; 44 School of Optometry and Vision Science, University of New South Wales, Sydney, Australia; 45 University of Rochester, School of Medicine and Dentistry, Rochester, NY USA; 46 University of Texas Southwestern Medical Center, Dallas, USA; 47 HelpMeSee, Instituto Mexicano de Oftalmologia, New York, USA; 48 University of Miami, Coral Gables, USA; 49 University of Utah, Salt Lake City, USA; 50 Nova Southeastern University College for Optometry, Fort Lauderdale, Florida USA; 51 African Vision Research Institute, University of KwaZulu-Natal (UKZN), Durban, South Africa; 52 Suraj Eye Instate, 559, New colony, Nagpur, India; 53 Institute of Optics and Optometry, University of Social Science, 121 Gdanska str, Lodz, 90-519 Poland; 54 Medicine & Health, University of New South Wales, Sydney, NSW Australia; 55 Centre for Public Health, Queens University Belfast, Northern Ireland, Belfast, UK; 56 John Hopkins Wilmer Eye Institute, Baltimore, USA; 57 1st Department of Ophthalmology, Medical School, Aristotle University of Thessaloniki, Ahepa Hospital, Thessaloniki, Greece; 58 University of Crete Medical School, Giofirakia, Greece; 59 Beijing Institute of Ophthalmology, Beijing Tongren Hospital, Capital Medical University, Beijing Ophthalmology and Visual Sciences Key Laboratory, Beijing, China; 60 Beijing Institute of Ophthamology, Beijing Tongren Eye Center, Beijing Tongren Hospital, Capital Medical University, Beijing, China; 61 Division of Ophthalmology, University of São Paulo, Ribeirão Preto, Brazil; 62 Institute of Molecular and Clinical Ophthalmology Basel, Basel, Switzerland; 63 Department of Ophthalmology, Heidelberg University, Mannheim, Germany; 64 Department of Ophthalmology and Visual Sciences, Federal University of São Paulo, São Paulo, Brazil; 65 Department of Ophthalmology, San Raffaele Scientific Institute, Milano, Italy; 66 Ophthalmology Department, CHU de Poitiers (Poitiers University Hospital), Poitiers, France; 67 National Institute of Health and Medical Research (INSERM), Poitiers, France; 68 Institute for Health Metrics and Evaluation, University of Washington, Seattle, WA USA; 69 Department of Health Metrics Sciences, School of Medicine, University of Washington, Seattle, WA USA; 70 School of Optometry and Vision Science, University of New South Wales, Sydney, NSW Australia; 71 Institute of Public Health, Charité Universitätsmedizin Berlin (Charité Medical University Berlin), Berlin, Germany; 72 Department of Clinical Governance and Quality Improvement, Aleta Wondo Hospital, Aleta Wondo, Ethiopia; 73 The Institute of Pharmaceutical Sciences (TIPS), Tehran University of Medical Sciences, Tehran, Iran; 74 School of Pharmacy, Tehran University of Medical Sciences, Tehran, Iran; 75 Department of Public Health, Wolkite University, Wolkite, Ethiopia; 76 Centre for Social Research in Health, University of New South Wales, Sydney, NSW Australia; 77 Quality and Systems Performance Unit, Cancer Institute NSW, Sydney, NSW Australia; 78 School of Public Health & Dept. of Community Medicine, Chitwan Medical College & Teaching Hospital, Bharatpur, Nepal; 79 Public Health Section, Himalayan Environment and Public Health Network (HEPHN), Chitwan, Nepal; 80 Department of Physiology, Government Institute of Medical Sciences, Greater Noida, India; 81 Department of Medical and Surgical Sciences and Advanced Technologies “G.F. Ingrassia”, University of Catania, Catania, Italy; 82 Department of Geography and Planning, Queen’s University, Kingston, ON Canada; 83 Department of Medical Biochemistry, Shaqra University, Shaqra, Saudi Arabia; 84 Ophthalmic Research Center, Shahid Beheshti University of Medical Sciences, Tehran, Iran; 85 Department of Ophthalmology, Shahid Beheshti University of Medical Sciences, Tehran, Iran; 86 Bascom Palmer Eye Institute, University of Miami, Miami, FL USA; 87 Evidence-Based Practice Center, Mayo Clinic Foundation for Medical Education and Research, Rochester, MN USA; 88 Department of Ophthalmology, University of Leipzig Medical Center, Leipzig, Germany; 89 Department of Ophthalmology, Charité Medical University Berlin, Berlin, Germany; 90 Institute of Health Research, University of Health and Allied Sciences, Ho, Ghana; 91 Center for Biotechnology and Microbiology, University of Swat, Swat, Pakistan; 92 Wilmer Eye Institute, Johns Hopkins University School of Medicine, Baltimore, MD USA; 93 Doheny Image Reading and Research Lab (DIRRL) - Doheny Eye Institute, University of California Los Angeles, Los Angeles, CA USA; 94 Department of Medicine, University of Thessaly, Volos, Greece; 95 Department of Pharmacology, All India Institute of Medical Sciences, Jodhpur, India; 96 All India Institute of Medical Sciences, Bhubaneswar, India; 97 School of Dentistry and Medical Sciences, Charles Sturt University, Orange, NSW Australia; 98 Health Management and Economics Research Center, Iran University of Medical Sciences, Tehran, Iran; 99 Department of Public Health, Jimma University, Jimma, Ethiopia; 100 Department of Public Health, Wachemo University, Hossana, Ethiopia; 101 Department of Immunology, Zanjan University of Medical Sciences, Zanjan, Iran; 102 Department of Health System and Health Economics, Bahir Dar University, Bahir Dar, Ethiopia; 103 Department of Forensic Medicine, Lumbini Medical College, Palpa, Nepal; 104 Epidemiology, Eyu-Ethiopia, Bahir dar, Ethiopia; 105 College of Medicine and Health Science, Bahir Dar University, Bahir dar, Ethiopia; 106 Department of Nursing, Debre Berhan University, Debre Berhan, Ethiopia; 107 Department of Nursing, University of Alberta, Edmonton, AB Canada; 108 Department of Nursing, Injibara University, Injibara, Ethiopia; 109 Department of Neurovascular Research, Nested Knowledge, Inc, Saint Paul, MN USA; 110 Faculty of Medicine, October 6 University, 6th of October City, Egypt; 111 School of Medicine, Isfahan University of Medical Sciences, Isfahan, Iran; 112 School of Public Affairs, Nanjing University of Science and Technology, Nanjing, China; 113 Department of Medical and Surgical Sciences and Advanced Technologies “GF Ingrassia”, University of Catania, Catania, Italy; 114 Miami Cancer Institute, Baptist Health South Florida, Miami, FL USA; 115 Heidelberg Institute of Global Health (HIGH), Heidelberg University, Heidelberg, Germany; 116 T.H. Chan School of Public Health, Harvard University, Boston, MA USA; 117 Department of Surgery, Jimma University, Jimma, Ethiopia; 118 Farabi Eye Hospital, Tehran University of Medical Sciences, Tehran, Iran; 119 School of Sport, Exercise and Health Sciences, Loughborough University, Loughborough, UK; 120 School of Public Health, Imperial College London, London, UK; 121 Department of Regulatory Affairs, NSF International, Ann Arbor, GA USA; 122 Department of Regulatory Affairs, Amarex Clinical Research, Germantown, MD USA; 123 Department of Public Health, North Dakota State University, Fargo, ND USA; 124 Global Health Neurology Lab, NSW Brain Clot Bank, Sydney, NSW Australia; 125 Department of Neurology and Neurophysiology, South West Sydney Local Heath District and Liverpool Hospital, Sydney, NSW Australia; 126 Medical Lab Technology, Chandigarh University, Mohali, India; 127 Department of Human Genetics and Molecular Medicine, Central University of Punjab, Bathinda, India; 128 Department of Health Behaviour, Environment and Social Medicine, Universitas Gadjah Mada (Gadjah Mada University), Sleman, Indonesia; 129 Center of Health and Behavior and Promotion, Universitas Gadjah Mada (Gadjah Mada University), Sleman, Indonesia; 130 Department of Medicine, Division of Clinical Epidemiology, McGill University, Montreal, QC Canada; 131 Centre for Outcomes Research and Evaluation, Research Institute of the McGill University Health Centre, Montreal, QC Canada; 132 Ophthalmology Department, Moorfields Eye Hospital NHS Foundation Trust, London, UK; 133 International Centre for Eye Health, London School of Hygiene & Tropical Medicine, London, UK; 134 Department of Biopharmaceutics and Clinical Pharmacy, The University of Jordan, Amman, Jordan; 135 Department of Basic Biomedical Sciences, University of Sharjah, Sharjah, United Arab Emirates; 136 Harvard Business School, Harvard University, Boston, MA USA; 137 School of Sciences, University of Minho, Braga, Portugal; 138 Association of Licensed Optometry Professionals, Linda-a-Velha, Portugal; 139 College of Public Health, Medical and Veterinary Sciences, James Cook University, Townsville, QLD Australia; 140 Public Health Department, University of Mataram, Mataram, Indonesia; 141 Temerty Faculty of Medicine, University of Toronto, Toronto, ON Canada; 142 Department of Community Medicine, Datta Meghe Institute of Medical Sciences, Sawangi, India; 143 Center for Biomedicine and Community Health, VNU-International School, Hanoi, Viet Nam; 144 Therapeutic and Diagnostic Technologies, Cooperativa de Ensino Superior Politécnico e Universitário (Polytechnic and University Higher Education Cooperative), Gandra, Portugal; 145 Institute for Research and Innovation in Health, University of Porto, Porto, Portugal; 146 Department of Addiction Medicine, Haukland University Hospital, Bergen, Norway; 147 Department of Global Public Health and Primary Care, University of Bergen, Bergen, Norway; 148 Ophthalmology Department, Carol Davila University of Medicine and Pharmacy, Bucharest, Romania; 149 Ophthalmology Department, Emergency University Hospital Bucharest, Bucuresti, Romania; 150 Department of Radiology, Tabriz University of Medical Sciences, Tabriz, Iran; 151 2nd University Ophthalmology Department, Aristotle University of Thessaloniki, Thessaloniki, Greece; 152 Ophthalmology Department, University of Thessaly, Thessaly, Greece; 153 Department of Radiology, Iran University of Medical Sciences, Tehran, Iran; 154 Tehran University of Medical Science, Tehran University of Medical Sciences, Tehran, Iran; 155 Epidemiology Branch, National Institute of Health, Durham, NC USA; 156 St Paul’s Eye Unit, Royal Liverpool University Hospital, Liverpool, UK; 157 Department of Ophthalmology, Aristotle University of Thessaloniki, Thessaloniki, Greece; 158 Department of Community Medicine, Chettinad Academy of Research and Education, Chennai, India; 159 Department of Human Physiology, University of Gondar, Gondar, Ethiopia; 160 Development of Research and Technology Center, Ministry of Health and Medical Education, Tehran, Iran; 161 Department of Ophthalmology and Visual Sciences, University of Michigan, Ann Arbor, MI USA; 162 Institute for Health Care Policy and Innovation, University of Michigan, Ann Arbor, MI USA; 163 Department of Epidemiology and Medical Statistics, University of Ibadan, Ibadan, Nigeria; 164 Faculty of Public Health, University of Ibadan, Ibadan, Nigeria; 165 Department of Biological Sciences, University of Medical Sciences, Ondo, Ondo Nigeria; 166 Biomedical Informatics and Medical Statistics Department, Alexandria University, Alexandria, Egypt; 167 Faculty of Medicine, University of Tripoli, Tripoli, Libya; 168 Department of Ophthalmology, University of California Los Angeles, Los Angeles, CA USA; 169 Department of Nursing, Wolkite University, Wolkite, Ethiopia; 170 Research Centre for Healthcare and Community, Coventry University, Coventry, UK; 171 Department of Oral Biology, The University of Lahore, Lahore, Pakistan; 172 Department of Nutrition and Dietetics, National University of Medical Sciences (NUMS), Rawalpindi, Pakistan; 173 School of Medicine, Tehran University of Medical Sciences, Tehran, Iran; 174 Endocrinology and Metabolism Research Institute, Non-Communicable Diseases Research Center (NCDRC), Tehran, Iran; 175 Department of Environmental Health Engineering, Isfahan University of Medical Sciences, Isfahan, Iran; 176 Department of Social medicine and epidemiology, Guilan University of Medical Sciences, Rasht, Iran; 177 University Eye Clinic, University of Genoa, Genoa, Italy; 178 Department of Nursing, Wollega University, Nekemte, Ethiopia; 179 Institute of Health sciences, Department of Public Health, Wollega University, Nekemte, Ethiopia; 180 Department of Mathematics, Imperial College London, London, UK; 181 Department of Ophthalmology, Isfahan University of Medical Sciences, Isfahan, Iran; 182 Emergency Department, Isfahan University of Medical Sciences, Isfahan, Iran; 183 Department of Biotechnological and Applied Clinical Sciences (DISCAB), University of L’Aquila, L’Aquila, Italy; 184 Department of Neuroscience, Hospital Santa Maria delle Croci, Ravenna, Italy; 185 Child Survival Unit, Centre for African Newborn Health and Nutrition, Ibadan, Nigeria; 186 Department of Community Medicine and Family Medicine, All India Institute of Medical Sciences, Nagpur, India; 187 Health Sciences department of Oncology Nursing, Haramaya University, Harar, Ethiopia; 188 Department of Midwifery, Adigrat University, Adigrat, Ethiopia; 189 Department of Environmental Health, Wollo University, Dessie, Ethiopia; 190 Department of Health, Policy Research Institute, Mekelle, Ethiopia; 191 Simon Fraser University, Mekelle, Ethiopia; 192 Department of Nursing, Aksum University, Aksum, Ethiopia; 193 Department of Nursing, Mekelle University, Mekelle, Ethiopia; 194 School of Public Health, Wolaita Sodo University, Sodo, Ethiopia; 195 Eye Research Center, Iran University of Medical Sciences, Tehran, Iran; 196 Ophthalmology Department, Tehran University of Medical Sciences, Tehran, Iran; 197 Department of Radiology, Mayo Clinic, Rochester, MN USA; 198 Health Systems and Policy Research Department, Indian Institute of Public Health, Gandhinagar, India; 199 Department of Genetics, Sana Institute of Higher Education, Sari, Iran; 200 Universal Scientific Education and Research Network (USERN), Kermanshah University of Medical Sciences, Kermanshah, Iran; 201 Toxicology Department, Shriram Institute for Industrial Research, Delhi, India; 202 School of Medicine, Deakin University, Geelong, VIC Australia; 203 Faculty of Medicine Health and Human Sciences, Macquarie University, Sydney, NSW Australia; 204 Faculty of Medicine, The University of Queensland, Brisbane, QLD Australia; 205 Department of Radiology, Massachusetts General Hospital, Boston, MA USA; 206 Obesity Research Center, Shahid Beheshti University of Medical Sciences, Tehran, Iran; 207 Research Institute for Endocrine Sciences, Shahid Beheshti University of Medical Sciences, Tehran, Iran; 208 Department of Ophthalmology, Thomas Jefferson University, Philadelphia, PA USA; 209 Ophthalmology, University of California San Diego, La Jolla, CA USA; 210 Brain and Behavioral Sciences Program, University of Georgia, Athens, GA USA; 211 Department of Pharmaceutical Technology, University of Dhaka, Dhaka, Bangladesh; 212 Department of Ophthalmology, Iran University of Medical Sciences, Karaj, Iran; 213 Social Determinants of Health Research Center, Shahid Beheshti University of Medical Sciences, Tehran, Iran; 214 Chapter of Addiction Medicine, University of Sydney, Sydney, NSW Australia; 215 Department of Epidemiology, Non-Communicable Diseases Research Center (NCDRC), Tehran, Iran; 216 School of Medicine, Shahid Beheshti University of Medical Sciences, Tehran, Iran; 217 Department of Pediatrics, Yonsei University College of Medicine, Seoul, South Korea; 218 Research Department, Electronic Medical Records for the Developing World, York, UK; 219 School of Social Sciences, The Apollo University, Chittoor, India; 220 Institute of Research and Development, Duy Tan University, Da Nang, Viet Nam; 221 Department of Computer Science, University of Human Development, Sulaymaniyah, Iraq; 222 Department of Psychology, Tsinghua University, Beijing, China; 223 International Master Program for Translational Science, Taipei Medical University, Taipei, Taiwan; 224 School of Health Systems and Public Health, University of Pretoria, Pretoria, South Africa; 225 Research and Development Unit, Biomedical Research Networking Center for Mental Health Network (CiberSAM), Sant Boi de Llobregat, Spain; 226 Faculty of Medicine, University of Versailles Saint-Quentin-en-Yvelines, Montigny-le-Bretonneux, France; 227 Department of Immunology, Kerman University of Medical Sciences, Kerman, Iran; 228 Department of Immunology, Rafsanjan University of Medical Sciences, Rafsanjan, Iran; 229 The World Academy of Sciences UNESCO, Trieste, Italy; 230 Shaanxi University of Technology, Hanzhong, China; 231 Department of Biochemistry, Government Medical College, Mysuru, India; 232 Zoonoses Research Center, Islamic Azad University, Karaj, Iran; 233 Department of Clinical Sciences, Jahrom University of Medical Sciences, Jahrom, Iran; 234 Department of Community Medicine, Manipal Academy of Higher Education, Mangalore, India; 235 Department of Economics, National Open University, Benin City, Nigeria; 236 Department of Environmental and Occupational Health, University of Gondar, Gondar, Ethiopia; 237 Social Determinants of Health Research Center, Gonabad University of Medical Sciences, Gonabad, Iran; 238 Manipal Institute of Management, Manipal Academy of Higher Education, Manipal, India; 239 Save Sight Institute, University of Sydney, Sydney, NSW Australia; 240 Sydney Eye Hospital, South Eastern Sydney Local Health District, Sydney, NSW Australia; 241 School of Health Professions and Human Services, Hofstra University, Hempstead, NY USA; 242 Department of Anesthesiology, Montefiore Medical Center, Bronx, NY USA; 243 Health Policy Research Center, Shiraz University of Medical Sciences, Shiraz, Iran; 244 International Research Center of Excellence, Institute of Human Virology Nigeria, Abuja, Nigeria; 245 Julius Centre for Health Sciences and Primary Care, Utrecht University, Utrecht, Netherlands; 246 Department of Public Health, Werabe University, Werabe, Ethiopia; 247 Department of Public Health, Jordan University of Science and Technology, Irbid, Jordan; 248 Amity Institute of Forensic Sciences, Amity University, Noida, India; 249 Department of Basic Medical Sciences, Yarmouk University, Irbid, Jordan; 250 Global Consortium for Public Health Research, Datta Meghe Institute of Higher Education and Research, Wardha, India; 251 Ophthalmology and Vision Science, Shahid Beheshti University of Medical Sciences, Tehran, Iran; 252 School of Traditional Chinese Medicine, Xiamen University Malaysia, Sepang, Malaysia; 253 School of Health Sciences, Kristiania University College, Oslo, Norway; 254 Department of International Health and Sustainable Development, Tulane University, New Orleans, LA USA; 255 Department of Nursing and Health Promotion, Oslo Metropolitan University, Oslo, Norway; 256 Independent Consultant, Jakarta, Indonesia; 257 San Juan de Dios Sanitary Park, Barcelona, Spain; 258 Department of Anthropology, Panjab University, Chandigarh, India; 259 Department of Health Policy and Strategy, Foundation for People-centric Health Systems, New Delhi, India; 260 SD Gupta School of Public Health, Indian Institute of Health Management Research University, Jaipur, India; 261 Department of Physiotherapy, Universitas Aisyiyah Yogyakarta, Yogyakarta, Indonesia; 262 Institute of Allied Health Sciences, National Cheng Kung University, Tainan, Taiwan; 263 Indian Council of Medical Research, New Delhi, India; 264 Chief Medical Office, HelpMeSee, New York, NY USA; 265 Mexican Institute of Ophthalmology, Queretaro, Mexico; 266 University of Medicine and Pharmacy at Ho Chi Minh City, Ho Chi Minh City, Viet Nam; 267 Independent Consultant, Ho Chi Minh City, Viet Nam; 268 College of Optometry, Nova Southeastern University, Fort Lauderdale, FL USA; 269 Department of Medical Science, Ajou University School of Medicine, Suwon, South Korea; 270 Department of Precision Medicine, Sungkyunkwan University, Suwon-si, South Korea; 271 Department of Family Medicine, University of Texas, Galveston, TX USA; 272 Lerner Research Institute, Cleveland Clinic, Cleveland, OH USA; 273 Department of Quantitative Health Science, Case Western Reserve University, Cleveland, OH USA; 274 Department of Ophthalmology, Tehran University of Medical Sciences, Tehran, Iran; 275 Department of Food Hygiene and Safety, Qazvin University of Medical Sciences, Qazvin, Iran; 276 Department of Internal Medicine, Dayanand Medical College and Hospital, Ludhiana, India; 277 Digestive Diseases Research Institute, Tehran University of Medical Sciences, Tehran, Iran; 278 Faculty of Humanities and Health Sciences, Curtin University, Malaysia, Sarawak Malaysia; 279 Jeffrey Cheah School of Medicine and Health Sciences, Monash University, Subang Jaya, Malaysia; 280 Department of Ophthalmology, Princess of Wales Hospital, Wales, UK; 281 School of Optometry and Vision Sciences, Cardiff University, Cardiff, UK; 282 Department of Public Health, Arba Minch University, Arbaminch, Ethiopia; 283 Department of Nursing, Arba Minch University, Arba Minch, Ethiopia; 284 University Centre Varazdin, University North, Varazdin, Croatia; 285 Department of Medical Physiology, Madda Walabu University, Bale-Goba, Ethiopia; 286 Medical School, Macquarie University, Sydney, NSW Australia; 287 Molecular Biology Unit, Sirius Training and Research Centre, Khartoum, Sudan; 288 Bio-Statistical and Molecular Biology Department, Sirius Training and Research Centre, Khartoum, Sudan; 289 University of Gondar, University of Gondar, Gondar, Ethiopia; 290 Department of Biostatistics, Shiraz University of Medical Sciences, Shiraz, Iran; 291 School of Health and Rehabilitation Sciences, The University of Queensland, Brisbane, QLD Australia; 292 Non-communicable Diseases Research Center, Tehran University of Medical Sciences, Tehran, Iran; 293 Iran University of Medical Sciences, Tehran, Iran; 294 Non-communicable Disease Research Center, Tehran University of Medical Sciences, Tehran, Iran; 295 Department of Medicine, Democritus University of Thrace, Alexandroupolis, Greece; 296 Discipline of Optometry, University of KwaZulu-Natal, Durban, South Africa; 297 College of Medicine and Public Health, Flinders University, Adelaide, SA Australia; 298 Department of Engineering, Western Sydney University, Sydney, NSW Australia; 299 University Institute of Public Health, The University of Lahore, Lahore, Pakistan; 300 Department of Dental Public Health, King Abdulaziz University, Jeddah, Saudi Arabia; 301 Department of Health Policy and Oral Epidemiology, Harvard University, Boston, MA USA; 302 Independent Consultant, Tehran, Iran; 303 Department of Internal Medicine, Ardabil University of Medical Science, Ardabil, Iran; 304 Department of Medical Laboratory Sciences, Adigrat University, Adigrat, Ethiopia; 305 Division of Cardiology, Massachusetts General Hospital, Boston, MA USA; 306 Department of Medical Engineering, University of South Florida, Tampa, FL USA; 307 Cardiovascular Research Department, Methodist Hospital, Merrillville, IL USA; 308 Department of Surgery, Danang Family Hospital, Danang, Viet Nam; 309 Department of General Medicine, University of Medicine and Pharmacy at Ho Chi Minh City, Ho Chi Minh City, Viet Nam; 310 International Islamic University Islamabad, Islamabad, Pakistan; 311 Microbiology and Molecular Genetics, The Women University Multan, Multan, Pakistan; 312 Department of Physiology, University of Benin, Edo, Nigeria; 313 Department of Physiology, Benson Idahosa University, Benin City, Nigeria; 314 Department of Veterinary Public Health and Preventive Medicine, University of Ilorin, Ilorin, Nigeria; 315 Department of Psychiatry and Behavioural Neurosciences, McMaster University, Hamilton, ON Canada; 316 Department of Psychiatry, University of Lagos, Lagos, Nigeria; 317 Department of Nursing Science, Bowen University, Iwo, Nigeria; 318 Department of Pharmacology and Therapeutics, University of Nigeria Nsukka, Enugu, Nigeria; 319 Department of Pharmacotherapy and Pharmaceutical Care, Medical University of Warsaw, Warsaw, Poland; 320 School of Medicine, Western Sydney University, Campbelltown, NSW Australia; 321 Department of Optometry and Vision Science, University of KwaZulu-Natal, KwaZulu-Natal, South Africa; 322 Laboratory of Public Health Indicators Analysis and Health Digitalization, Moscow Institute of Physics and Technology, Dolgoprudny, Russia; 323 Department of Medicine, University of Ibadan, Ibadan, Nigeria; 324 Department of Medicine, University College Hospital, Ibadan, Ibadan, Nigeria; 325 Department of Forensic Medicine and Toxicology, Kasturba Medical College, Mangalore, Mangalore, India; 326 School of Medicine, Iran University of Medical Sciences, Tehran, Iran; 327 Research Department, Nepal Health Research Council, Kathmandu, Nepal; 328 Research Department, Public Health Research Society Nepal, Kathmandu, Nepal; 329 Global Health Governance Programme, University of Edinburgh, Edinburgh, UK; 330 School of Dentistry, University of Leeds, Leeds, UK; 331 Department of Internal Medicine, Advent Health, Palm Coast, FL USA; 332 Department of Hospital Medicine, Sound Physicians, Palm Coast, FL USA; 333 Department of Genetics, Yale University, New Haven, CT USA; 334 Department of Development Studies, International Institute for Population Sciences, Mumbai, India; 335 Department of Statistics and Econometrics, Bucharest University of Economic Studies, Bucharest, Romania; 336 Medical School, Pham Ngoc Thach University of Medicine, Ho Chi Minh City, Viet Nam; 337 Department of Neonatology, Case Western Reserve University, Cleveland, OH USA; 338 Department of Community Medicine and Family Medicine, All India Institute of Medical Sciences, Jodhpur, India; 339 Department of Health Sciences, Cihan University Sulaimaniya, Sulaymaniyah, Iraq; 340 Cihan University Sulaimaniya Research Center (CUSRC), Sulaymaniyah, Iraq; 341 Sina Trauma and Surgery Research Center, Tehran University of Medical Sciences, Tehran, Iran; 342 Manipal TATA Medical College, Manipal Academy of Higher Education, Manipal, India; 343 Department of Population Science and Human Resource Development, University of Rajshahi, Rajshahi, Bangladesh; 344 Department of Community Medicine, Mahatma Gandhi Medical College and Research Institute, Puducherry, India; 345 Department of Medicine, Jinnah Sindh Medical University, Karachi, Pakistan; 346 Department of Health Behaviour, Environment, and Social Medicine, Gadjah Mada University, Yogyakarta, Indonesia; 347 Department Biological Sciences, King Abdulaziz University, Jeddah, Egypt; 348 Department of Protein Research, Research and Academic Institution, Alexandria, Egypt; 349 Department of Labour, Directorate of Factories, Government of West Bengal, Kolkata, India; 350 Faculty of Medicine, Gonabad University of Medical Sciences, Gonabad, Iran; 351 Infectious Diseases Research Center, Gonabad University of Medical Sciences, Gonabad, Iran; 352 Department of Epidemiology, Shahid Beheshti University of Medical Sciences, Tehran, Iran; 353 College of Medicine, University of Sharjah, Sharjah, United Arab Emirates; 354 Multidisciplinary Laboratory Foundation University School of Health Sciences (FUSH), Foundation University, Islamabad, Pakistan; 355 International Center of Medical Sciences Research (ICMSR), Islamabad, Pakistan; 356 Ophthalmic Epidemiology Research Center, Shahid Beheshti University of Medical Sciences, Tehran, Iran; 357 Faculty of Medicine, Bioscience and Nursing, MAHSA University, Selangor, Malaysia; 358 Interdisciplinary Research Centre in Biomedical Materials (IRCBM), COMSATS Institute of Information Technology, Lahore, Pakistan; 359 Research Center for Immunodeficiencies, Tehran University of Medical Sciences, Tehran, Iran; 360 Sharjah Institute of Medical Sciences, University of Sharjah, Sharjah, United Arab Emirates; 361 Clinical Sciences Department, University of Sharjah, Sharjah, United Arab Emirates; 362 Applied Biomedical Research Center, Mashhad University of Medical Sciences, Mashhad, Iran; 363 Biotechnology Research Center, Mashhad University of Medical Sciences, Mashhad, Iran; 364 University of Warwick, Coventry, UK; 365 Institute for Employment Research, Nuremberg, Germany; 366 Medical Laboratory, Azad University of Medical Sciences, Tehran, Iran; 367 Minimally Invasive Surgery Research Center, Iran University of Medical Sciences, Tehran, Iran; 368 Advanced Therapy Medicinal Products Department, Royan Institution, Tehran, Iran; 369 Faculty of Pharmacy, Mansoura University, Mansoura, Egypt; 370 School of Public Health, Taipei Medical University, Taipei, Taiwan; 371 Department of Anatomy, Ras Al Khaimah Medical and Health Sciences University, Ras Al Khaimah, United Arab Emirates; 372 Department of Entomology, Ain Shams University, Cairo, Egypt; 373 Medical Ain Shams Research Institute (MASRI), Ain Shams University, Cairo, Egypt; 374 Department of Pharmacology and Research, All India Institute of Medical Sciences, Jodhpur, India; 375 Indira Gandhi Medical College and Research Institute, Puducherry, India; 376 Department of Public Health Sciences, University of North Carolina at Charlotte, Charlotte, NC USA; 377 Market Access Division, Bayer, Istanbul, Türkiye; 378 Comprehensive Ophthalmology, NOOR Eye Training Center, International Assistance Misison, Kabul, Afghanistan; 379 Research Department, Online Research Club, Nagasaki, Japan; 380 Faculty of Dentistry, AIMST University, Bedong, Malaysia; 381 Department of Medicine and Surgery, Government Doon Medical College, Dehradun, India; 382 National Heart, Lung, and Blood Institute, National Institute of Health, Rockville, MD USA; 383 Medical Research Center, Kateb University, Kabul, Afghanistan; 384 Institute of Molecular Biology and Biotechnology (IMBB), The University of Lahore, Lahore, Pakistan; 385 Research Centre for Health Sciences (RCHS), The University of Lahore, Lahore, Pakistan; 386 Centre of Medical and Bio Allied Health Sciences Research, Ajman University, Ajman, United Arab Emirates; 387 Independent Consultant, Karachi, Pakistan; 388 Department of Ophthalmology, Harvard Medical School, Boston, MA USA; 389 Ophthalmic Research Center (ORC), Shahid Beheshti University of Medical Sciences, Tehran, Iran; 390 National Institute of Infectious Diseases, Tokyo, Japan; 391 Department of Veterinary Public Health and Preventive Medicine, Usmanu Danfodiyo University, Sokoto, Sokoto, Nigeria; 392 Department of Medical-Surgical Nursing, Mazandaran University of Medical Sciences, Sari, Iran; 393 Department of Nursing and Health Sciences, Flinders University, Adelaide, SA Australia; 394 Unit of Basic Medical Sciences, University of Khartoum, Khartoum, Sudan; 395 Department of Medical Microbiology and Infectious Diseases, Erasmus University, Rotterdam, Netherlands; 396 Family, Health Promotion, and Life Course Department, Pan American Health Organization, Washington, DC USA; 397 School of Medicine, University of Alabama at Birmingham, Birmingham, AL USA; 398 Medicine Service, US Department of Veterans Affairs (VA), Birmingham, AL USA; 399 Department of Radiodiagnosis, All India Institute of Medical Sciences, Bathinda, India; 400 Department of Ophthalmology, Hywel Dda University Health Board, Llanelli, UK; 401 Directive Board, Associação de Profissionais Licenciados de Optometria (Association of Licensed Optometry Professionals), Linda-a-Velha, Portugal; 402 Division of Community Medicine, International Medical University, Kuala Lumpur, Malaysia; 403 Federal Research Institute for Health Organization and Informatics of the Ministry of Health (FRIHOI), Moscow, Russia; 404 School of Nursing and Midwifery, Debre Berhan University, Debre Berehan, Ethiopia; 405 Faculty of Public Health, Universitas Sam Ratulangi, Manado, Indonesia; 406 Medical School, University of Crete, Heraklion, Greece; 407 Institute of Soil and Environmental Sciences, University of Agriculture, Faisalabad, Faisalabad, Pakistan; 408 Medical Genomics Research Department, King Abdullah International Medical Research Center, Riyadh, Saudi Arabia; 409 Department of Life Sciences, University of Management and Technology, Lahore, Pakistan; 410 Clinical Cancer Research Center, Milad General Hospital, Tehran, Iran; 411 Department of Microbiology, Islamic Azad University, Tehran, Iran; 412 Department of Community Medicine, Rajarata University of Sri Lanka, Anuradhapura, Sri Lanka; 413 Department of Ophthalmology Research, Queen Mamohato Memorial Hospital, Maseru, Lesotho; 414 Ophthalmology Unit, Bahir Dar University, Bahir dar, Ethiopia; 415 Department of Cancer Epidemiology and Prevention Research, Alberta Health Services, Calgary, AB Canada; 416 Department of Oncology, University of Calgary, Calgary, AB Canada; 417 Department of Health Management, Süleyman Demirel Üniversitesi (Süleyman Demirel University), Isparta, Türkiye; 418 Department of Pediatrics, Kyung Hee University, Seoul, South Korea; 419 Department of Neuropsychopharmacology, National Center of Neurology and Psychiatry, Kodaira, Japan; 420 Department of Public Health, Juntendo University, Tokyo, Japan; 421 Macquarie Medical School, Macquarie University, Sydney, NSW Australia; 422 Department of Bioengineering and Therapeutic Sciences, University of California San Francisco, San Francisco, CA USA; 423 Addictology Department, Russian Medical Academy of Continuous Professional Education, Moscow, Russia; 424 College of Traditional Chinese Medicine, Hebei University, Baoding, China; 425 Department of Ophthalmology, Iran University of Medical Sciences, Tehran, Iran; 426 Department of Biochemistry and Pharmacogenomics, Medical University of Warsaw, Warsaw, Poland

**Keywords:** Epidemiology, Retinal diseases

## Abstract

**Background:**

We aimed to update estimates of global vision loss due to age-related macular degeneration (AMD).

**Methods:**

We did a systematic review and meta-analysis of population-based surveys of eye diseases from January, 1980, to October, 2018. We fitted hierarchical models to estimate the prevalence of moderate and severe vision impairment (MSVI; presenting visual acuity from <6/18 to 3/60) and blindness ( < 3/60) caused by AMD, stratified by age, region, and year.

**Results:**

In 2020, 1.85 million (95%UI: 1.35 to 2.43 million) people were estimated to be blind due to AMD, and another 6.23 million (95%UI: 5.04 to 7.58) with MSVI globally. High-income countries had the highest number of individuals with AMD-related blindness (0.60 million people; 0.46 to 0.77). The crude prevalence of AMD-related blindness in 2020 (among those aged ≥ 50 years) was 0.10% (0.07 to 0.12) globally, and the region with the highest prevalence of AMD-related blindness was North Africa/Middle East (0.22%; 0.16 to 0.30). Age-standardized prevalence (using the GBD 2019 data) of AMD-related MSVI in people aged ≥ 50 years in 2020 was 0.34% (0.27 to 0.41) globally, and the region with the highest prevalence of AMD-related MSVI was also North Africa/Middle East (0.55%; 0.44 to 0.68). From 2000 to 2020, the estimated crude prevalence of AMD-related blindness decreased globally by 19.29%, while the prevalence of MSVI increased by 10.08%.

**Conclusions:**

The estimated increase in the number of individuals with AMD-related blindness and MSVI globally urges the creation of novel treatment modalities and the expansion of rehabilitation services.

## Introduction

Age-related macular degeneration (AMD) is an acquired, degenerative disorder affecting the macula of older adults [1]. The disease is characterised by an array of features in the macular region, such as drusen (deposits that accumulate between the retinal pigment epithelium -RPE- and the Bruch’s membrane), RPE cell depigmentation and proliferation, detachment of the RPE, loss of RPE cells and subretinal choroidal neovascularization [1], is categorized into a non-exudative (or “dry”) stage and an exudative (or ‘wet’) stage. Older age, genetic background, family history, European ancestry, and smoking are considered risk factors for the development and progression of the disease [1].

Significant progress has been made in the treatment of the exudative stage of AMD over the last two decades due to the clinical introduction of intraocular injections of anti-vascular endothelial growth factor (VEGF) drugs [2]. Still, AMD has remained a challenge in global eye healthcare due to its relatively high prevalence in older adults and since most cases of the non-exudative stage, the most common form of AMD, cannot effectively be treated yet. AMD has a high importance as a cause of vision impairment and blindness [3], with sequels such as a reduction of the quality of life [4] and economic impact due to loss of productivity [5].

Although AMD-related blindness is a growing global problem due to population aging, its importance is not homogeneous across world regions. In countries with a higher cataract surgical coverage (usually high-income countries), chronic eye conditions such as AMD account for a relatively more significant proportion of blindness [6].

To contribute to the World Health Organization (WHO) World Report on Vision [7] and the implementation of the recommendations generated [8], The Vision Loss Expert Group (VLEG) of the Global Burden of Disease (GBD) Study calculated estimates of the leading causes of vision impairment and blindness, like AMD [3, 9–11]. Such estimates are essential for monitoring, action planning, and advocacy [6]. In this systematic review, we aimed to update estimates of the global vision loss burden due to AMD, presenting estimates for 2020, temporal changes, and distribution by sex and world region.

## Methods

The data was arranged following a review of published population-focused studies on vision impairment and blindness by the VLEG. This review included studies published between Jan 1, 1980, and Oct 1, 2018, incorporating grey literature. After title and abstract screening, abstracts were sent to regional VLEG committees, where at least three ophthalmic epidemiologists independently scored studies for quality against inclusion criteria. They were asked to review and rate each study with a score of 1 (clearly a representative population-based study using a comprehensive methodology), 2 (questionable representativeness and/or inadequately described or low-quality methodology), or 3 (definitely neither, warranting exclusion). Reviewers were asked to give their rationale for a rating of 2 or 3. A threshold for inclusion/exclusion was decided based on the average score of each study. Relevant studies from this review were combined with data from Rapid Assessment of Avoidable Blindness (RAAB) studies by VLEG. Data was also sourced from the US National Health and Nutrition Examination Survey 2007-2008 and the WHO Study on Global Ageing and Adult Health (SAGE Wave 1 2007-2010), contributed by the GBD team. A total of 252 studies contributed data on age-related macular degeneration and are grouped by geographical region in the Appendix. More detailed methods are published elsewhere [3, 10] and discussed in brief as follows.

VLEG pinpointed 137 studies and pulled data from 70 studies in their 2010 review and 67 additional studies in their 2014–18 review. Most of these studies were national or subnational cross-sectional surveys. VLEG also arranged to produce 5-year age-segregated RAAB data from the RAAB repository (www.raab.world). To qualify, studies met specific criteria: vision acuity data must be gathered through a test chart compatible with the Snellen scale, and the sample must represent the population. Subjective reports of vision loss were not included. The criteria for vision loss was defined by the International Classification of Diseases 11th edition as employed by WHO. It was based on the vision in the better eye upon presentation. Moderate vision loss was defined as a visual acuity of 6/60 or better but less than 6/18, severe vision loss as a visual acuity of 3/60 or better but less than 6/60, and blindness as a visual acuity of less than 3/60 or less than 10° visual field around central fixation (although the visual field definition was rarely used in population-based eye surveys).

We split the original data into several datasets, creating separate envelopes for each degree of vision loss (mild, moderate, and severe) and blindness. This data was then fed into a meta-regression tool designed by the Institute for Health Metrics and Evaluation (IHME) known as MR-BRT (meta-regression; Bayesian; regularised; trimmed) [12]. The benchmark for each severity level was presenting vision impairment.

When possible, data about uncorrected refractive errors were pulled straight from the data sources. If not, they were calculated by subtracting the best-corrected vision impairment from presenting vision impairment prevalence at each severity level. Other causes were factored into the best-corrected estimates for each level of vision impairment.

Our models for distance vision impairment and blindness were based on the most commonly reported causes found in the literature, and the minimum age for inclusion of data on AMD was 45 years. We created estimates of MSVI and blindness specific to location, year, age, and sex using Disease Modelling Meta-Regression (Dismod-MR) 2.1 [13]. Its data processing steps have been outlined elsewhere [3]. Briefly, Dismod-MR 2.1 models were run for all vision impairment by severity (moderate, severe, blindness) regardless of cause and, separately, for MSVI and blindness due to each modelled cause of vision impairment. Then, models of MSVI due to specific causes were split into moderate and severe estimates using the ratio of overall prevalence in the all-cause moderate presenting vision impairment and severe presenting vision impairment models. Next, prevalence estimates for all causes by severity were scaled to the models of all-cause prevalence by severity. This produced final estimates by age, sex, year, and location for each cause of vision impairment by severity. We age-standardised our estimates using the GBD standard population [14]. Hierarchical logistic regressions with mixed effects were applied using the R package RStanArm to assess the prevalence of blindness independently and MSVI across various country-age groups, structured within a five-tiered hierarchy. The blindness model was based on 270 studies, while the MSVI model included 245 studies, acknowledging that a single study might span multiple countries or years. This framework spanned 187 countries, grouped into 21 subregions and further into seven broader regions, culminating in an analysis of global-level effects. Data on blindness and MSVI due to AMD were presented by seven super-regions (Southeast Asia/East Asia/Oceania, Central Europe/Eastern Europe/Central Asia, High-income, Latin America and Caribbean, North Africa, and the Middle East, South Asia, and Sub-Saharan Africa) and globally. Data on other causes of vision impairment and blindness will be presented in separate publications.

## Results

In 2020, 1.85 million (all ages; 95% uncertainty interval (UI): 1.35 to 2.43 million) people were estimated to be blind due to AMD, with 664,000 (472,000 to 894,000) males and 1,185,000 (876,000 to 1,545,000) females affected (Tables 1, 2). AMD-related MSVI affected 6.23 million (95%UI: 5.04 to 7.58) individuals worldwide, among them 2,747,000 (2,207,000 to 3,377,000) males and 2,743,000 (2,202,000 to 3,371,000) females (Tables 1, 3).

**Table 1:** Number of people (mean [95% UI]) with blindness (presenting visual acuity <3/60) or MSVI (presenting visual acuity <6/18, ≥3/60) due to AMD, the age-standardized prevalence (%) in people of all ages and aged ≥50 years (mean [95% UI]), and the percentage of all blindness or MSVI attributed to AMD (95% UI) in world regions in 2020.

World Region	2020, total population (‘000 s) (all ages)	2020, total population (‘000 s) (aged ≥ 50 years)	Blindness due to AMD in 2020				MSVI due to AMD in 2020			
			Number of people (‘000 s) with blindness in 2020 (all ages)	Number of people (‘000 s) with blindness in 2020 (aged ≥ 50 years)	Age-standardized prevalence of AMD blindness in 2020 (aged ≥ 50 years)	Percentage contribution by AMD to all causes of blindness in 2020 (aged ≥50 years)	Number of people (‘000 s) with MSVI in 2020 (all ages)	Number of people (‘000 s) with MSVI in 2020 (aged ≥ 50 years)	Age-standardized prevalence of AMD MSVI in 2020 (aged ≥ 50 years)	Percentage contribution by AMD to all causes of MSVI in 2020 (aged ≥50 years)
Global	7,890,000	1.898,000	1850 (1349–2434)	1841 (1340–2423)	0.10 (0.08–0.14)	4.30 (3.14–5.66)	6231 (5042–7587)	6221 (5029–7573)	0.34 (0.27–0.41)	2.11 (1.71–2.57)
Southeast Asia, East Asia, and Oceania	2,192,710	664,195	495 (342–680)	493 (339–673)	0.08 (0.06–0.11)	3.29 (2.27–4.51)	2762 (2211–3379)	2758 (2209–3376)	0.46 (0.37–0.56)	3.33 (2.66–4.07)
Central Europe, Eastern Europe, and Central Asia	417,291	137,758	62 (43–84)	62 (43–83)	0.04 (0.03–0.06)	4.43 (3.07–5.95)	228 (182–282)	227 (182–282)	0.16 (0.13–0.19)	1.27 (1.01–1.57)
Highincome	1,087,856	423,872	596 (456–769)	595 (455–768)	0.11 (0.08–0.14)	19.82 (15.17–25.60)	739 (584–917)	738 (584–917)	0.14 (0.11–0.17)	2.38 (1.88–2.95)
Latin America and Caribbean	601,551	136,738	71 (49–97)	71 (49–97)	0.05 (0.04–0.07)	1.95 (1.35–2.66)	333 (270–407)	332 (269–406)	0.25 (0.21–0.31)	1.36 (1.10–1.67)
North Africa and Middle East	631,727	105,278	195 (136–264)	194 (136–264)	0.22 (0.16–0.30)	6.31 (4.43–8.55)	493 (391–614)	492 (390–613)	0.55 (0.44–0.68)	2.26 (1.79–2.81)
South Asia	1,841,435	321,354	297 (200–423)	295 (199–421)	0.10 (0.07–0.15)	2.49 (1.68–3.55)	1220 (972–1510)	1217 (968–1507)	0.42 (0.34–0.51)	1.27 (1.01–1.57)
Sub-Saharan Africa	1,114,806	108,805	131 (92–178)	131 (91–178)	0.15 (0.11–0.20)	2.58 (1.81–3.51)	454 (360–565)	452 (358–564)	0.50 (0.40–0.61)	2.22 (1.76–2.77)

**Table 2:** Number of males and females with blindness (presenting visual acuity <3/60), and the age-standardized prevalence (% [95% UI]) of blindness due to AMD (all ages and people aged ≥50 years) in 2020.

World Region	Total Population	Total number of AMD blindness and aged-standardized AMD blindness in 2020 (all ages)				Total Number of AMD blindness and aged-standardized AMD blindness in people aged 50+ years in 2020			
	2020, total population (‘000 s)	Number of males(‘000 s) with AMD blindness in 2020	Number of females (‘000 s) with AMD blindness in 2020	Age standardized prevalence of blindness in males in 2020	Age standardized prevalence of blindness in females in 2020	Number of males(‘000 s) (50+ years) with AMD blindness in 2020	Number of females(‘000 s) (50+ years) with AMD blindness in 2020	Age standardized prevalence of blindness in males in 2020	Age standardized prevalence of blindness in females in 2020
Global	7,890,000	664 (472–894)	1185 (876–1545)	0.0 (0.0–0.0)	0.0 (0.0–0.0)	660 (469–890)	1180 (871–1540)	0.1 (0.1–0.1)	0.1 (0.1–0.2)
Southeast Asia, East Asia, and Oceania	2,192,710	185 (128–257)	309 (214–424)	0.0 (0.0–0.0)	0.0 (0.0–0.0)	184 (126–254)	307 (212–422)	0.1 (0.1–0.1)	0.1 (0.1–0.1)
Central Europe, Eastern Europe, and Central Asia	417,291	20 (14–28)	42 (29–56)	0.0 (0.0–0.0)	0.0 (0.0–0.0)	20 (14–27)	41 (29–56)	0.0 (0.0–0.0)	0.1 (0.0–0.1)
Highincome	1,087,856	168 (124–222)	427 (327–543)	0.0 (0.0–0.0)	0.0 (0.0–0.0)	168 (124–221)	427 (327–542)	0.1 (0.1–0.1)	0.1 (0.1–0.2)
Latin America and Caribbean	601,551	23 (15–32)	48 (33–65)	0.0 (0.0–0.0)	0.0 (0.0–0.0)	23 (15–32)	47 (33–65)	0.0 (0.0–0.1)	0.1 (0.1–0.1)
North Africa and Middle East	631,727	77 (53–105)	117 (81–159)	0.0 (0.0–0.1)	0.1 (0.0–0.1)	77 (53–104)	116 (81–158)	0.2 (0.1–0.3)	0.3 (0.2–0.4)
South Asia	1,841,435	142 (95–199)	155 (105–221)	0.0 (0.0–0.0)	0.0 (0.0–0.0)	141 (94–198)	154 (104–219)	0.1 (0.1–0.2)	0.1 (0.1–0.2)
Sub-Saharan Africa	1,114,806	46 (32–62)	85 (59–115)	0.0 (0.0–0.0)	0.0 (0.0–0.1)	45 (32–62)	84 (59–114)	0.1 (0.1–0.2)	0.2 (0.1–0.2)

**Table 3:** Number of males and females with AMD MSVI, and the age-standardized prevalence (% [95% uncertainty intervals (UIs)]) of AMD MSVI (all ages and people aged ≥50 years) in 2020.

World Region	Total Population	Total number of AMD MSVI and aged-standardized AMD MSVI in 2020 (all ages)				Total number of AMD MSVI and aged-standardized AMD MSVI in people aged 50+ years in 2020			
	2020, total population (‘000 s)	Number of males(‘000 s) with AMD MSVI in 2020	Number of females (‘000 s) with AMD MSVI in 2020	Age standardized prevalence of MSVI in males in 2020	Age standardized prevalence of MSVI in females in 2020	Number of females(‘000 s) (50+ years) with AMD MSVI in 2020	Number of females(‘000 s) (50+ years) with AMD MSVI in 2020	Age standardized prevalence of MSVI in males in 2020	Age standardized prevalence of MSVI in females in 2020
Global	7,890,000	2747 (2207–3377)	3483 (2823–4236)	0.1 (0.1–0.1)	0.1 (0.1–0.1)	2743 (2202–3371)	3478 (2816–4226)	0.3 (0.3–0.4)	0.4 (0.3–0.4)
Southeast Asia, East Asia, and Oceania	2,192,710	1231 (982–1501)	1530 (1224–1865)	0.1 (0.1–0.1)	0.1 (0.1–0.1)	1230 (981–1500)	1528 (1223–1862)	0.4 (0.4–0.5)	0.5 (0.4–0.6)
Central Europe, Eastern Europe, and Central Asia	417,291	75 (60–94)	152 (121–188)	0.0 (0.0–0.0)	0.0 (0.0–0.1)	75 (60–94)	152 (121–188)	0.1 (0.1–0.2)	0.2 (0.1–0.2)
High–income	1,087,856	294 (232–363)	445 (350–554)	0.0 (0.0–0.0)	0.0 (0.0–0.0)	293 (232–362)	444 (350–553)	0.1 (0.1–0.2)	0.2 (0.1–0.2)
Latin America and Caribbean	601,551	137 (110–168)	196 (159–240)	0.1 (0.0–0.1)	0.1 (0.1–0.1)	136 (109–168)	195 (158–239)	0.2 (0.2–0.3)	0.3 (0.2–0.3)
North Africa and Middle East	631,727	234 (185–295)	258 (204–320)	0.1 (0.1–0.1)	0.1 (0.1–0.2)	234 (185–295)	258 (204–320)	0.5 (0.4–0.7)	0.6 (0.5–0.7)
South Asia	1,841,435	571 (453–704)	649 (518–802)	0.1 (0.1–0.1)	0.1 (0.1–0.1)	570 (452–703)	647 (517–800)	0.4 (0.3–0.5)	0.4 (0.4–0.5)
SubSaharan Africa	1,114,806	202 (160–253)	251 (198–313)	0.1 (0.1–0.1)	0.1 (0.1–0.1)	202 (160–252)	250 (198–313)	0.5 (0.4–0.6)	0.5 (0.4–0.6)

High-income countries (0.60 million people; 0.46 to 0.77) accounted for the highest number of individuals with AMD-related blindness per world region, whereas the lowest number of individuals with presenting blindness due to AMD per world region was found in Central Europe / Eastern Europe / Central Asia combined (0.06 million people; 0.04 to 0.08) and Latin America and the Caribbean (0.07 million people; 0.50 to 0.1) (Table 1).

The crude prevalence of AMD-related blindness in those aged ≥ 50 years in 2020 was estimated as 0.10% (0.07 to 0.12) globally. The regions with the highest prevalence of blindness due to AMD were North Africa/Middle East, 0.22% (0.16 to 0.30) and Sub-Saharan Africa, 0.15% (0.11 to 0.20) (Supplementary Fig. 1). Age-standardized estimated prevalence of MSVI due to AMD in those aged ≥ 50 years in 2020 was 0.34% (0.27 to 0.41) globally, and the regions with the highest prevalence of MSVI due to AMD were also North Africa/Middle East (0.55%; 0.44 to 0.68) and Sub-Saharan Africa (0.50%; 0.40 to 0.61) (Table 1).

From 2000 to 2020, the estimated crude prevalence of blindness due to AMD decreased globally by 19.3% (19.6 to 19.0%), with a wide variety between regions, from -32.6% (-32.9 to -32.3%) in Southeast Asia, East Asia, and Oceania to +1.3% (0.9 to 1.7%) in Latin America and the Caribbean, the only region which showed an increase (Table 4). On the other hand, over the same period, the estimated crude prevalence of MSVI due to AMD increased globally by 10.1% (9.8 to 10.3%), with changes varying from −9.3% (−9.5 to −9.0%) in North Africa and Middle East to +7.2% (6.9 to 7.5%) in Southeast Asia, East Asia, and Oceania (Table 5). Figure 2 shows the estimated crude prevalence of MSVI due to AMD in 2020.

**Table 4:** Percentage change in crude prevalence, number of people affected, and age-standardised prevalence of AMD blindness (presenting visual acuity <3/60) in adults age 50 years and older between 2000 and 2020 by world super region.

	Crude Prevalence			Number of Cases (‘000 s)			Age standardized prevalence		
World Region	Male (%, 95% UI)	Female (%, 95% UI)	Both (%, 95% UI)	Male (%, 95% UI)	Female (%, 95% UI)	Both (%, 95% UI)	Male (%, 95% UI)	Female (%, 95% UI)	Both (%, 95% UI)
Global	−18.8 (−19.1 to −18.5)	−19.5 (−19.8 to −19.3)	−19.3 (−19.6 to −19.0)	43.3 (42.7 to 43.8)	41.4 (40.9 to 41.9)	42.1 (41.6 to 42.5)	−22.2 (−22.5 to −21.9)	−21.7 (−22.0 to −21.5)	−22.6 (−22.8 to −22.3)
High-income	3.9 (3.5 to 4.2)	−4.2 (−4.5 to −3.9)	−3.1 (−3.4 to −2.8)	52.8 (52.3 to 53.3)	33.5 (33.1 to 33.8)	38.4 (38.0 to 38.8)	−12.8 (−13.1 to −12.5)	−14.4 (−14.7 to −14.2)	−15.8 (−16.0 to −15.5)
Central Europe, Eastern Europe, and Central Asia	1.9 (1.5 to 2.3)	−10.0 (−10.4 to −9.7)	−6.9 (−7.3 to −6.6)	32.2 (31.7 to 32.7)	11.3 (10.9 to 11.8)	17.4 (17.0 to 17.9)	−5.0 (−5.3 to −4.6)	−15.1 (−15.4 to −14.7)	−12.7 (−13.0 to −12.3)
Latin America and Caribbean	−2.6 (−3.0 to −2.2)	2.5 (2.1 to 2.9)	1.3 (0.9 to 1.7)	92.0 (91.2 to 92.8)	109.9 (109.0 to 110.7)	103.6 (102.8 to104.5)	−6.6 (−7.0 to −6.3)	−3.7 (−4.1 to −3.3)	−4.1 (−4.5 to −3.7)
North Africa and Middle East	−23.8 (−24.1 to −23.5)	−18.4 (−18.7 to −18.0)	−20.6 (−20.9 to −20.3)	58.4 (57.8 to 59.0)	69.9 (69.2 to 70.6)	65.1 (64.5 to 65.8)	−17.4 (−17.7 to −17.1)	−16.4 (−16.7 to −16.1)	−16.4 (−16.7 to −16.0)
South Asia	−28.3 (−28.7 to −28.0)	−32.7 (−32.9 to −32.4)	−30.5 (−30.8 to −30.2)	35.2 (34.7 to 35.8)	36.2 (35.6 to 36.8)	35.7 (35.1 to 36.3)	−28.5 (−28.8 to −28.2)	−34.1 (−34.4 to −33.9)	−31.4 (−31.7 to −31.1)
Southeast Asia, East Asia, and Oceania	−33.8 (−34.1 to −33.5)	−32.2 (−32.5 to −31.9)	−32.6 (−32.9 to −32.3)	30.9 (30.4 to 31.4)	38.3 (37.8 to 38.9)	35.5 (34.9 to 36.0)	−36.3 (−36.5 to −36.0)	−32.5 (−32.8 to −32.3)	−34.1 (−34.3 to −33.8)
Sub-Saharan Africa	−11.9 (−12.3 to −11.6)	−12.4 (−12.7 to −12.0)	−11.2 (−11.6 to −10.9)	55.8 (55.2 to 56.4)	70.0 (69.3 to 70.6)	64.7 (64.1 to 65.4)	−7.4 (−7.8 to −7.0)	−6.8 (−7.2 to −6.5)	−6.6 (−7.0 to −6.3)

**Table 5:** Percentage change in crude prevalence, number of people affected, and age-standardised prevalence of AMD MSVI (presenting visual acuity <6/18, ≥3/60) and in adults aged 50 years and older between 2000 and 2020 by world super region.

	Crude Prevalence			Number of Cases (‘000 s)			Age standardized prevalence		
World Region	Male (%, 95% UI)	Female (%, 95% UI)	Both (%, 95% UI)	Male (%, 95% UI)	Female (%, 95% UI)	Both (%, 95% UI)	Male (%, 95% UI)	Female (%, 95% UI)	Both (%, 95% UI)
Global	9.8 (9.6 to 10.1)	10.3 (10.0 to 10.6)	10.1 (9.8 to 10.3)	93.7 (93.2 to 94.2)	93.8 (93.3 to 94.2)	93.7 (93.3 to 94.2)	6.5 (6.3 to 6.8)	10.5 (10.2 to 10.8)	8.7 (8.4 to 8.9)
High-income	12.9 (12.6 to 13.2)	2.1 (1.8 to 2.4)	5.6 (5.3 to 5.9)	66.1 (65.7 to 66.6)	42.2 (41.8 to 42.6)	50.8 (50.4 to 51.2)	−3.2 (−3.4 to −2.9)	−5.0 (−5.2 to −4.7)	−4.8 (−5.0 to −4.6)
Central Europe, Eastern Europe, and Central Asia	6.9 (6.6 to 7.2)	7.1 (6.8 to 7.4)	6.6 (6.3 to 6.9)	38.7 (38.3 to 39.1)	32.5 (32.2t o 32.9)	34.5 (34.2 to 34.9)	−0.0 (−0.3 to 0.2)	4.3 (4.0 to 4.6)	2.6 (2.4 to 2.9)
Latin America and Caribbean	3.7 (3.4 to 3.9)	7.8 (7.6 to 8.1)	6.3 (6.0 to 6.5)	104.3 (103.8 to 104.8)	120.7 (120.2 to 121.3)	113.7 (113.2 to 114.2)	0.0 (−0.2 to 0.3)	3.9 (3.7 to 4.2)	2.4 (2.1 t o2.6)
North Africa and Middle East	−11.9 (−12.2 to −11.7)	−6.7 (−7.0 to −6.5)	−9.3 (−9.5 to −9.0)	83.0 (82.5 to 83.5)	94.1 (93.6 to 94.6)	88.6 (88.1 to 89.1)	−4.6 (−4.8 to−4.3)	−3.2 (−3.4 to −2.9)	−3.8 (−4.1 to −3.6)
South Asia	5.8 (5.5 to 6.0)	2.1 (1.8 to 2.3)	4.0 (3.8 to 4.3)	99.6 (99.1 to 100.1)	106.4 (105.8 to 106.9)	103.1 (102.6 to 103.7)	0.4 (0.1 to 0.6)	−3.2 (−3.5 to −3.0)	−1.5 (−1.7 to −1.2)
Southeast Asia, East Asia, and Oceania	7.4 (7.2 to 7.7)	6.8 (6.5 to 7.0)	7.2 (6.9 to 7.5)	112.5 (111.9 to 113.0)	117.8 (117.2 to 118.3)	115.4 (114.8 to 115.9)	5.7 (5.4 to 5.9)	7.3 (7.0 to 7.5)	6.4 (6.2 to 6.7)
Sub-Saharan Africa	−8.4 (−8.7 to −8.2)	−0.5 (−0.8 to −0.2)	−4.2 (−4.4 to −3.9)	62.0 (61.6 to 62.4)	93.1 (92.5 to 93.6)	77.9 (77.4 to 78.3)	−2.5 (−2.7 to −2.3)	4.1 (3.8 to 4.4)	0.7 (0.5 to 1.0)

## Discussion

In 2020, AMD ranked second among the causes of irreversible blindness globally [3]. From 2000 to 2020, there was an estimated decrease in the prevalence of AMD-related blindness in all regions except Latin America and the Caribbean and an increase in the prevalence of AMD-related MSVI in all regions except North Africa, Middle East, and Sub-Saharan Africa, with wide discrepancies between regions. The global population growth and increasing life expectancy can explain the increasing number of individuals with AMD-related vision loss. Yet, the divergences in AMD prevalence and vision impairment necessitate extensive research to unravel the complex interplay of genetic factors, lifestyle choices, and access to healthcare services.

The prevalence of blindness and MSVI due to AMD is higher in older age groups, and countries with a growing life expectancy should take this information into account for better health service planning. But considering the current barriers to accessing AMD treatment and rehabilitation services in many regions, even a minor increase in absolute numbers might put pressure on the already overloaded public health systems. The reduced availability of ophthalmic services during the Covid-19 pandemic [15] and the economic impact that might be seen during the post-pandemic years can increase barriers in managing chronic eye conditions like AMD.

Dealing with AMD from a public health perspective is a complex task. First, although a trained ophthalmologist efficiently performs diagnosis, the availability and distribution of eye care professionals remain an issue in many parts of the world [16]. If well connected to public health initiatives, the growing use of telemedicine and artificial intelligence in eye care will likely reduce the percentage of those with undiagnosed AMD. Second, its first-line treatment, intravitreal anti-VEGF injection, can be expensive; its effect lasts only a few months and is only indicated for the wet form and, more recently, geographic atrophy [17]. And finally, those with MSVI and blindness due to AMD would benefit from rehabilitation services, and their availability is also scarce [18]. The integration of education for those at a higher risk for developing AMD diagnosis, treatment, and rehabilitation, using a people-centred approach [7], could reduce the burden of AMD. Ideally, addressing AMD from a public health perspective demands a life course, people-centered approach recognizing the importance of early interventions and managing AMD co-morbidities. Preventative measures, such as anti-smoking campaigns targeted at adolescents and adults, could significantly impact the prevalence of AMD in the older population, given the well-established link between smoking and the progression of AMD.

In the healthcare continuum, involving primary care providers (PCPs) in AMD care could alleviate some current issues, especially in low-resource scenarios. They can serve as the initial touchpoint for patient education and promote awareness of AMD risk factors and early symptoms. For example, PCPs could play a crucial role in guiding patients through lifestyle changes that mitigate the risk of AMD, such as advocating smoking cessation, reinforcing the need for regular check-ups for those with diagnosed AMD or at a higher risk of having it, and potentially managing portable fundus cameras that could send images for remote ophthalmologists, or reinforcing the importance of compliance with follow-ups and treatment, and monitoring co-morbidities.

In addition to preventive care, there is a critical need to address the broader spectrum of challenges faced by individuals with AMD, particularly as they often experience other ocular and extraocular health issues. For instance, hearing impairment is a common co-morbidity that can compound the difficulties faced by those with vision loss, intensifying feelings of isolation and hindering effective communication. Moreover, individuals with vision impairment are at an increased risk of falls, which can lead to further physical injury and a decline in their quality of life [6]. Depression is another concern, with the loss of visual function significantly impacting mental health and the overall well-being of affected individuals [6].

The non-physician administration of anti-VEGF injections could be explored as a strategy to decentralize treatment, lowering costs and increasing access, while ensuring the safety and efficacy of such approaches through proper training and oversight [19]. Another option to be considered is conducting the injections within the same day of consultation, and same-day bilateral intravitreal injections to avoid multiple visits [20–22].

In the realm of age-related macular degeneration (AMD) treatments, emerging therapies like Pegcetacoplan and gene therapy offer renewed hope. Pegcetacoplan was recently the first drug approved by the Food and Drug Administration to treat geographic atrophy, the advanced stage of dry AMD known for its relentless progression of retinal cell degeneration and consequent permanent vision loss [17]. This novel drug specifically inhibits the C3 component of the immune system’s complement pathway, a system implicated in the exacerbation of GA. In a recently published clinical trial, intravitreal injections of Pegcetacoplan could successfully slow GA progression. However, no differences in visual acuity between eyes injected with either Pegcetacoplan or sham injections were found [17].

Gene therapy presents a frontier approach in AMD treatment by introducing genetic material into cells to correct abnormal genes, suppress harmful gene expression, or produce beneficial proteins. This can involve various strategies, from replacing defective genes with functional ones, silencing genes contributing to disease progression to introducing new genes that could stop disease progression or repair damaged retinal tissue. Initial clinical trials have shed light on gene therapy’s capacity for offering a sustained therapeutic effect, potentially simplifying treatment regimens by reducing the need for frequent injections, which are the current standard of care for wet AMD. Despite these promising developments, gene therapy for AMD remains in the experimental phase, and ongoing research is focused on overcoming hurdles related to precise delivery methods, ensuring lasting benefits, and establishing safety protocols [23].

Strengths of the present study included the number of population-based data accessed and used, analysis of trends in the causes of blindness and MSVI, incorporation of nonlinear age trends and accounting for data that were not reported by age, and systematic quantitative analysis and reporting of uncertainty. Among the limitations of the present study, we can cite the shortage of data in some countries/regions and information on the burden of AMD in ethnic minorities, such as indigenous people. In some countries, population-based data were either collected sub nationally or more than a decade ago, and estimates may not represent the current reality of a given country. Also, most analysed studies used the Rapid Assessment of Cataract Surgical Services or RAAB methodologies, which may underestimate posterior pole diseases, such as AMD. The evolution of population-based studies, especially those employing advanced imaging technologies like fundus photography, should be elaborated to reflect how these methodologies can refine the accuracy of AMD prevalence studies [24].

Policymakers and the academic community should consider that AMD’s social and economic impact is expected to increase substantially due to population growth and ageing [6]. Since population growth rates and life expectancies differ across regions, the burden of AMD will vary globally. Different from cataract and uncorrected refractive errors, the leading causes of blindness and vision impairment, respectively, the burden of AMD can only be alleviated if significant advances in research are made. Developing novel, cost-effective treatment modalities ideally restoring sight, and adopting a life course approach integrating all levels of care is paramount for managing AMD for the next decades.

## Summary

### What was known before:

- Based on the Global Burden of Disease Study 2010, the Vision Loss Expert Group (VLEG) estimated that there were 2.1 million people blind due to macular diseases.

### What this study adds:

- In 2020, 1.85 million (95%UI: 1.35 to 2.43 million) people were blind due to AMD, and another 6.23 million (95%UI: 5.04 to 7.58) presented with MSVI globally.
- From 2000 to 2020, there was a reduction in the crude prevalence of age-related macular degeneration (AMD)-related blindness globally and an increase in AMD-related moderate and severe vision impairment.

### Supplementary information

Supplemental Figure 1

Supplemental Figure 2

Supplemental Table 1

Supplementary material

Supplementary Appendix

## Data Availability

The data that support the findings of this study are not openly available due to reasons of sensitivity and are available from the coordinator of the Vision Loss Expert Group (Professor Rupert Bourne; rb@rupertbourne.co.uk) upon reasonable request. Data are located in controlled access data storage at Anglia Ruskin University.

## Appendix: Contributions by Authors

GBD 2019 Blindness and Vision Impairment Collaborators

Providing data or critical feedback on data sources

Yohannes Habtegiorgis Abate, Tadele Girum Girum Adal, Kishor Adhikari, Antonella Agodi, Williams Agyemang-Duah, Fares Alahdab, Syed Shujait Shujait Ali, Louay Almidani, Sofia Androudi, Jalal Arabloo, Alessandro Arrigo, Seyyed Shamsadin Athari, Desta Debalkie Atnafu, Alok Atreya, Yared Asmare Aynalem, Zewdu Bishaw Aynalem, Ahmed Y Azzam, Sara Bagherieh, Martina Barchitta, Mainak Bardhan, Till Winfried Bärnighausen, Nebiyou Simegnew Bayileyegn, Ahmet Begde, Babak Behnam, Akshaya Srikanth Bhagavathula, Sonu Bhaskar, Gurjit Kaur Bhatti, Jasvinder Singh Bhatti, Bagas Suryo Bintoro, Rupert R. A. Bourne, Tasanee Braithwaite, Paul Svitil Briant, Florentino Luciano Caetano dos Santos, Muthia Cenderadewi, Vijay Kumar Chattu, Dinh-Toi Chu, Maria Vittoria Cicinelli, Natália Cruz-Martins, Xiaochen Dai, Maedeh Dastmardi, Nikolaos Dervenis, Vinoth Gnana Chellaiyan Devanbu, Joshua R Ehrlich, Michael Ekholuenetale, Temitope Cyrus Ekundayo, Iman El Sayed, Ambaw Abebaw Emrie, Adeniyi Francis Fagbamigbe, Hossein Farrokhpour, Ali Fatehizadeh, Alireza Feizkhah, Arthur G Fernandes, Lorenzo Ferro Desideri, Seth Flaxman, Kayode Raphael Fowobaje, João M Furtado, Tilaye Gebru Gebi, Brhane Gebremariam, Molalegn Mesele Gesese, Fariba Ghassemi, Sherief Ghozy, Mahaveer Golechha, Pouya Goleij, Sapna Gupta, Veer Bala Gupta, Vivek Kumar Gupta, Teklehaimanot Gereziher Haile, Arvin Haj-Mirzaian, Sung Hwi Hong, Praveen Hoogar, Mehdi Hosseinzadeh, Chengxi Hu, Hong-Han Huynh, Chidozie C D Iwu, Mihajlo Jakovljevic, Shubha Jayaram, Jost B Jonas, Charity Ehimwenma Joshua, Gebisa Guyasa Kabito, Laleh R Kalankesh, Himal Kandel, Gbenga A Kayode, Shemsu Kedir, Yousef Saleh Khader, Himanshu Khajuria, Mahalaqua Nazli Khatib, Yun Jin Kim, Adnan Kisa, Sezer Kisa, Soewarta Kosen, Kewal Krishan, Chandrakant Lahariya, Tri Laksono, Dharmesh Kumar Lal, Trang Diep Thanh Le, Munjae Lee, Seung Won Lee, Nicolas Leveziel, Stephen S Lim, Xuefeng Liu, Razzagh Mahmoudi, Kashish Malhotra, Roy Rillera Marzo, Andrea Maugeri, Tesfahun Mekene Meto, Soheil Mohammadi, Ali H Mokdad, Mohammad Ali Moni, Maryam Moradi, Admir Mulita, Kovin S Naidoo, Ganesh R Naik, Shumaila Nargus, Zuhair S Natto, Biswa Prakash Nayak, Dang H Nguyen, Hien Quang Nguyen, Phat Tuan Nguyen, Van Thanh Nguyen, Robina Khan Niazi, Ogochukwu Janet Nzoputam, Ismail A Odetokun, Andrew T Olagunju, Matthew Idowu Olatubi, Obinna E Onwujekwe, Uchechukwu Levi Osuagwu, Mayowa O Owolabi, Jagadish Rao Padubidri, Jay Patel, Shrikant Pawar, Arokiasamy Perianayagam, Hoang Tran Pham, Fakher Rahim, Vafa Rahimi-Movaghar, Ahmed Mustafa Rashid, Annisa Utami Rauf, Elrashdy Moustafa Mohamed Redwan, Serge Resnikoff, Zahra Saadatian, Siamak Sabour, Basema Saddik, Umar Saeed, Sare Safi, Sher Zaman Safi, Narjes Saheb Sharif-Askari, Joseph W Sakshaug, Vijaya Paul Samuel, Abdallah M Samy, Monika Sawhney, Mete Saylan, Sayed Mansoor Sediqi, Yashendra Sethi, Allen Seylani, Jaffer Shah, Masood Ali Shaikh, Muhammad Aaqib Shamim, Maryam Shayan, Aminu Shittu, Jasvinder A Singh, Paramdeep Singh, Chandrashekhar T Sreeramareddy, Jaimie D Steinmetz, Ian Tapply, Hugh R Taylor, Guesh Mebrahtom Tsegay, Saif Ullah, Muhammad Umair, Sahel Valadan Tahbaz, Theo Vos, Dong Keon Yon, Naohiro Yonemoto, Mikhail Sergeevich Zastrozhin, and Magdalena Zielińska.

Developing methods or computational machinery

Alessandro Arrigo, Desta Debalkie Atnafu, Ahmed Y Azzam, Akshaya Srikanth Bhagavathula, Rupert R. A. Bourne, Paul Svitil Briant, Kaleb Coberly, Xiaochen Dai, Maedeh Dastmardi, Michael Ekholuenetale, Mehdi Emamverdi, Ayesha Fahim, Ali Fatehizadeh, Arthur G Fernandes, Seth Flaxman, Sherief Ghozy, Mehdi Hosseinzadeh, Hong-Han Huynh, Jost B Jonas, Charity Ehimwenma Joshua, Mahalaqua Nazli Khatib, Adnan Kisa, Chandrakant Lahariya, Nicolas Leveziel, Razzagh Mahmoudi, Ali H Mokdad, Mohammad Ali Moni, Admir Mulita, Hien Quang Nguyen, Phat Tuan Nguyen, Van Thanh Nguyen, Michal Ordak, Jagadish Rao Padubidri, Hoang Tran Pham, Premkumar Ramasubramani, Umar Saeed, Abdallah M Samy, Monika Sawhney, Tabassom Sedighi, Jaimie D Steinmetz, Ian Tapply, Hugh R Taylor, Muhammad Umair, Theo Vos, and Peng Zheng.

Providing critical feedback on methods or results

Yohannes Habtegiorgis Abate, Mohammad Abdollahi, Tadele Girum Girum Adal, Isaac Yeboah Addo, Kishor Adhikari, Antonella Agodi, Aqeel Ahmad, Hamid Ahmadieh, Hooman Ahmadzadeh, Fares Alahdab, Ahmad Samir Alfaar, Robert Kaba Alhassan, Syed Shujait Shujait Ali, Louay Almidani, Sofia Androudi, Anayochukwu Edward Anyasodor, Jalal Arabloo, Alessandro Arrigo, Mubarek Yesse Ashemo, Seyyed Shamsadin Athari, Desta Debalkie Atnafu, Alok Atreya, Melese Kitu Ayalew, Yared Asmare Aynalem, Zewdu Bishaw Aynalem, Ahmed Y Azzam, Sara Bagherieh, Ruhai Bai, Martina Barchitta, Mainak Bardhan, Till Winfried Bärnighausen, Nebiyou Simegnew Bayileyegn, Fatemeh Bazvand, Ahmet Begde, Babak Behnam, Akshaya Srikanth Bhagavathula, Sonu Bhaskar, Gurjit Kaur Bhatti, Jasvinder Singh Bhatti, Bagas Suryo Bintoro, Rupert R. A.  Bourne, Tasanee Braithwaite, Paul Svitil Briant, Katrin Burkart, Yasser Bustanji, Florentino Luciano Caetano dos Santos, Vera L A Carneiro, Muthia Cenderadewi, Vijay Kumar Chattu, Dinh-Toi Chu, Natália Cruz-Martins, Omid Dadras, Xiaochen Dai, Ana Maria Dascalu, Mohsen Dashti, Anna Dastiridou, Maedeh Dastmardi, Xinlei Deng, Nikolaos Dervenis, Vinoth Gnana Chellaiyan Devanbu, Mengistie Diress, Shirin Djalalinia, Michael Ekholuenetale, Temitope Cyrus Ekundayo, Iman El Sayed, Muhammed Elhadi, Mehdi Emamverdi, Ambaw Abebaw Emrie, Adeniyi Francis Fagbamigbe, Ayesha Fahim, Umar Farooq, Hossein Farrokhpour, Ali Fatehizadeh, Alireza Feizkhah, Arthur G Fernandes, Lorenzo Ferro Desideri, Getahun Fetensa, Bikila Regassa Feyisa, Florian Fischer, Seth Flaxman, Ali Forouhari, Matteo Foschi, Kayode Raphael Fowobaje, João M Furtado, Aravind P Gandhi, Tilaye Gebru Gebi, Miglas W Gebregergis, Mesfin Gebrehiwot, Brhane Gebremariam, Molalegn Mesele Gesese, Khalil Ghasemi Falavarjani, Fariba Ghassemi, Sherief Ghozy, Mahaveer Golechha, Sapna Gupta, Veer Bala Gupta, Vivek Kumar Gupta, Teklehaimanot Gereziher Haile, Semira Goitom Hailu, Arvin Haj-Mirzaian, Aram Halimi, Shahin Hallaj, Billy Randall Hammond, Ikramul Hasan, Hamidreza Hasani, Hossein Hassanian-Moghaddam, Mahsa Heidari-Foroozan, Sung Hwi Hong, Praveen Hoogar, Mehdi Hosseinzadeh, Chengxi Hu, Hong-Han Huynh, Mustapha Immurana, Chidozie C D Iwu, Louis Jacob, Mihajlo Jakovljevic, Shubha Jayaram, Mohammad Jokar, Jost B Jonas, Nitin Joseph, Charity Ehimwenma Joshua, Gebisa Guyasa Kabito, Laleh R Kalankesh, Sagarika Kamath, Himal Kandel, Ibraheem M Karaye, Gbenga A Kayode, Shemsu Kedir, Yousef Saleh Khader, Himanshu Khajuria, Moawiah Mohammad Khatatbeh, Mahalaqua Nazli Khatib, Zahra Khorrami, Yun Jin Kim, Adnan Kisa, Sezer Kisa, Ai Koyanagi, Kewal Krishan, Chandrakant Lahariya, Tri Laksono, Dharmesh Kumar Lal, Van Charles Lansingh, Trang Diep Thanh Le, Munjae Lee, Seung Won Lee, Wei-Chen Lee, Nicolas Leveziel, Stephen S Lim, Xuefeng Liu, Alireza Mahmoudi, Razzagh Mahmoudi, Kashish Malhotra, Vahid Mansouri, Roy Rillera Marzo, Andrea Maugeri, Colm McAlinden, Tesfahun Mekene Meto, Abera M Mersha, Tomislav Mestrovic, Ephrem Tesfaye Mihretie, Mehdi Mirzaei, Prasanna Mithra, Nouh Saad Mohamed, Soheil Mohammadi, Abdulwase Mohammed, Ali H Mokdad, Hossein Molavi Vardanjani, Mohammad Ali Moni, Fateme Montazeri, Maryam Moradi, Ahmed Nuru Muhamed, Admir Mulita, Ganesh R Naik, Shumaila Nargus, Zuhair S Natto, Biswa Prakash Nayak, Mohammad Negaresh, Hadush Negash, Seyed Aria Nejadghaderi, Dang H Nguyen, Hien Quang Nguyen, Phat Tuan Nguyen, Van Thanh Nguyen, Robina Khan Niazi, Ogochukwu Janet Nzoputam, Ismail A Odetokun, Andrew T Olagunju, Matthew Idowu Olatubi, Obinna E Onwujekwe, Michal Ordak, Uchechukwu Levi Osuagwu, Nikita Otstavnov, Mayowa O Owolabi, Jagadish Rao Padubidri, Parsa Panahi, Ashok Pandey, Shahina Pardhan, Jay Patel, Shrikant Pawar, Arokiasamy Perianayagam, Ionela-Roxana Petcu, Hoang Tran Pham, Ibrahim Qattea, Pankaja Raghav Raghav, Fakher Rahim, Vafa Rahimi-Movaghar, Mosiur Rahman, Premkumar Ramasubramani, Ahmed Mustafa Rashid, Annisa Utami Rauf, Elrashdy Moustafa Mohamed Redwan, Serge Resnikoff, Nazila Rezaei, Priyanka Roy, Zahra Saadatian, Siamak Sabour, Basema Saddik, Umar Saeed, Sare Safi, Sher Zaman Safi, Narjes Saheb Sharif-Askari, Joseph W Sakshaug, Mohamed A Saleh, Yoseph Leonardo Samodra, Vijaya Paul Samuel, Abdallah M Samy, Monika Sawhney, Mete Saylan, Sayed Mansoor Sediqi, Yashendra Sethi, Jaffer Shah, Samiah Shahid, Masood Ali Shaikh, Muhammad Aaqib Shamim, Maryam Shayan, Mika Shigematsu, Aminu Shittu, Seyed Afshin Shorofi, Emmanuel Edwar Siddig, Juan Carlos Silva, Jasvinder A Singh, Paramdeep Singh, Eirini Skiadaresi, Chandrashekhar T Sreeramareddy, Vladimir I Starodubov, Ian Tapply, Birhan Tsegaw Taye, Jansje Henny Vera Ticoalu, Guesh Mebrahtom Tsegay, Saif Ullah, Muhammad Umair, Sahel Valadan Tahbaz, Theo Vos, Nuwan Darshana Wickramasinghe, Guadie Sharew Wondimagegn, Arzu Yiğit, Dong Keon Yon, Naohiro Yonemoto, Yuyi You, Mikhail Sergeevich Zastrozhin, Hanqing Zhao, and Magdalena Zielińska.

Drafting the work or revising it critically for important intellectual content

Yohannes Habtegiorgis Abate, Mohammad Abdollahi, Tadele Girum Girum Adal, Isaac Yeboah Addo, Prerna Agarwal, Antonella Agodi, Hamid Ahmadieh, Hooman Ahmadzadeh, Fares Alahdab, Ahmad Samir Alfaar, Robert Kaba Alhassan, Syed Shujait Shujait Ali, Louay Almidani, Abhishek Anil, Anayochukwu Edward Anyasodor, Jalal Arabloo, Alessandro Arrigo, Seyyed Shamsadin Athari, Desta Debalkie Atnafu, Alok Atreya, Melese Kitu Ayalew, Ahmed Y Azzam, Sara Bagherieh, Martina Barchitta, Mainak Bardhan, Till Winfried Bärnighausen, Maurizio Battaglia Parodi, Ahmet Begde, Akshaya Srikanth Bhagavathula, Sonu Bhaskar, Gurjit Kaur Bhatti, Jasvinder Singh Bhatti, Marina G Birck, Rupert R. A. Bourne, Tasanee Braithwaite, Paul Svitil Briant, Yasser Bustanji, Florentino Luciano Caetano dos Santos, Muthia Cenderadewi, Vijay Kumar Chattu, Dinh-Toi Chu, Maria Vittoria Cicinelli, Natália Cruz-Martins, Ana Maria Dascalu, Anna Dastiridou, Maedeh Dastmardi, Nikolaos Dervenis, Joshua R Ehrlich, Michael Ekholuenetale, Iman El Sayed, Muhammed Elhadi, Mehdi Emamverdi, Ambaw Abebaw Emrie, Adeniyi Francis Fagbamigbe, Ayesha Fahim, Ali Fatehizadeh, Arthur G Fernandes, Getahun Fetensa, Florian Fischer, Seth Flaxman, Ali Forouhari, Matteo Foschi, João M Furtado, Aravind P Gandhi, Tilaye Gebru Gebi, Miglas W Gebregergis, Mesfin Gebrehiwot, Brhane Gebremariam, Gebreamlak Gebremedhn Gebremeskel, Yibeltal Yismaw Gela, Fariba Ghassemi, Sherief Ghozy, Sapna Gupta, Veer Bala Gupta, Vivek Kumar Gupta, Teklehaimanot Gereziher Haile, Aram Halimi, Shahin Hallaj, Billy Randall Hammond, Ikramul Hasan, Hamidreza Hasani, Hong-Han Huynh, Mustapha Immurana, Chidozie C D Iwu, Louis Jacob, Abdollah Jafarzadeh, Mihajlo Jakovljevic, Shubha Jayaram, Jost B Jonas, Nitin Joseph, Charity Ehimwenma Joshua, Gebisa Guyasa Kabito, Sagarika Kamath, Himal Kandel, Hengameh Kasraei, Gbenga A Kayode, Shemsu Kedir, Yousef Saleh Khader, Himanshu Khajuria, Moawiah Mohammad Khatatbeh, Mahalaqua Nazli Khatib, Adnan Kisa, Sezer Kisa, Ai Koyanagi, Kewal Krishan, Chandrakant Lahariya, Van Charles Lansingh, Janet L Leasher, Nicolas Leveziel, Xuefeng Liu, Razzagh Mahmoudi, Vahid Mansouri, Roy Rillera Marzo, Andrea Maugeri, Colm McAlinden, Tesfahun Mekene Meto, Tomislav Mestrovic, Ephrem Tesfaye Mihretie, Mehdi Mirzaei, Prasanna Mithra, Nouh Saad Mohamed, Soheil Mohammadi, Abdulwase Mohammed, Ali H Mokdad, Hossein Molavi Vardanjani, Mohammad Ali Moni, Fateme Montazeri, Maryam Moradi, Parsa Mousavi, Kovin S Naidoo, Shumaila Nargus, Zuhair S Natto, Biswa Prakash Nayak, Mohammad Negaresh, Hadush Negash, Seyed Aria Nejadghaderi, Dang H Nguyen, Hien Quang Nguyen, Phat Tuan Nguyen, Van Thanh Nguyen, Robina Khan Niazi, Mamoona Noreen, Ogochukwu Janet Nzoputam, Ismail A Odetokun, Andrew T Olagunju, Matthew Idowu Olatubi, Obinna E Onwujekwe, Michal Ordak, Uchechukwu Levi Osuagwu, Nikita Otstavnov, Mayowa O Owolabi, Jagadish Rao Padubidri, Shahina Pardhan, Jay Patel, Venkata Suresh Patthipati, Shrikant Pawar, Arokiasamy Perianayagam, Ionela-Roxana Petcu, Hoang Tran Pham, Ibrahim Qattea, Pankaja Raghav Raghav, Fakher Rahim, Vafa Rahimi-Movaghar, Mohammad Hifz Ur Rahman, Premkumar Ramasubramani, Ahmed Mustafa Rashid, Elrashdy Moustafa Mohamed Redwan, Serge Resnikoff, Nazila Rezaei, Priyanka Roy, Siamak Sabour, Basema Saddik, Umar Saeed, Amene Saghazadeh, Fatemeh Saheb Sharif-Askari, Amirhossein Sahebkar, Saina Salahi, Sarvenaz Salahi, Vijaya Paul Samuel, Abdallah M Samy, Aswini Saravanan, Monika Sawhney, Mete Saylan, Siddharthan Selvaraj, Yashendra Sethi, Allen Seylani, Jaffer Shah, Samiah Shahid, Moyad Jamal Shahwan, Muhammad Aaqib Shamim, Mika Shigematsu, Aminu Shittu, Seyed Afshin Shorofi, Emmanuel Edwar Siddig, Jasvinder A Singh, Paramdeep Singh, Eirini Skiadaresi, Raúl A R C Sousa, Chandrashekhar T Sreeramareddy, Vladimir I Starodubov, Birhan Tsegaw Taye, Miltiadis K Tsilimbaris, Saif Ullah, Muhammad Umair, Sahel Valadan Tahbaz, Theo Vos, Nuwan Darshana Wickramasinghe, Guadie Sharew Wondimagegn, Lin Yang, Arzu Yiğit, Dong Keon Yon, Naohiro Yonemoto, Mikhail Sergeevich Zastrozhin, Makan Ziafati, and Magdalena Zielińska.

Managing the estimation or publications process

Rupert R. A. Bourne, João M Furtado, Ali H Mokdad, and Theo Vos

Vision Loss Expert Group of the Global Burden of Disease Study

Providing data or critical feedback on data sources

Alessandro Arrigo, Maurizio Battaglia Parodi, Mukharram M Bikbov, Rupert R A Bourne, Tasanee Braithwaite, Alain Bron, Ching-Yu Cheng, Maria Vittoria Cicinelli, Nathan Congdon, Monte A Del Monte, Joshua R Ehrlich, Arthur Fernandes, Seth Flaxman, Tim Fricke, David Friedman, João M Furtado, Gus Gazzard, M Elizabeth Hartnett, Jost B Jonas, Rim Kahloun, John H Kempen, Moncef Khairallah, Rohit C Khanna, Judy E Kim, Van Charles Lansingh, Janet Leasher, Nicolas Leveziel, Kovin S Naidoo, Vinay Nangia, Michal Nowak, Konrad Pesudovs, Tunde Peto, Pradeep Ramulu, Serge Resnikoff, Tabassom Sedighi, Ian Tapply, Hugh Taylor, Fotis Topouzis, Miltiadis Tsilimbaris, Ya Xing Wang, Ningli Wang

Developing methods or computational machinery

Rupert R A Bourne, Jost B Jonas, Ian Tapply

Providing critical feedback on methods or results

Alessandro Arrigo, Mukharram M Bikbov, Rupert R A Bourne, Tasanee Braithwaite, Monte A Del Monte, David Friedman, João M Furtado, M Elizabeth Hartnett, Jost B Jonas, Rim Kahloun, John H Kempen, Konrad Pesudovs, Serge Resnikoff, Ian Tapply, Ningli Wang

Drafting the work or revising it critically for important intellectual content

Alessandro Arrigo, Mukharram M Bikbov, Rupert R A Bourne, Tasanee Braithwaite, Nathan Congdon, Monte A Del Monte, João M Furtado, M Elizabeth Hartnett, Jost B Jonas, Janet Leasher, Konrad Pesudovs

Managing the estimation or publications process

Rupert R A Bourne, João M Furtado, Jost B Jonas
