# Supplementary figures and images for: Global estimates on the number of people blind or visually impaired by age-related macular degeneration: a meta-analysis from 2000 to 2020
Source: Eye (Lond). 2024 Jul 4;38(11):2070–82. doi: 10.1038/s41433-024-03050-z (PMC11269688; doi:10.1038/s41433-024-03050-z)

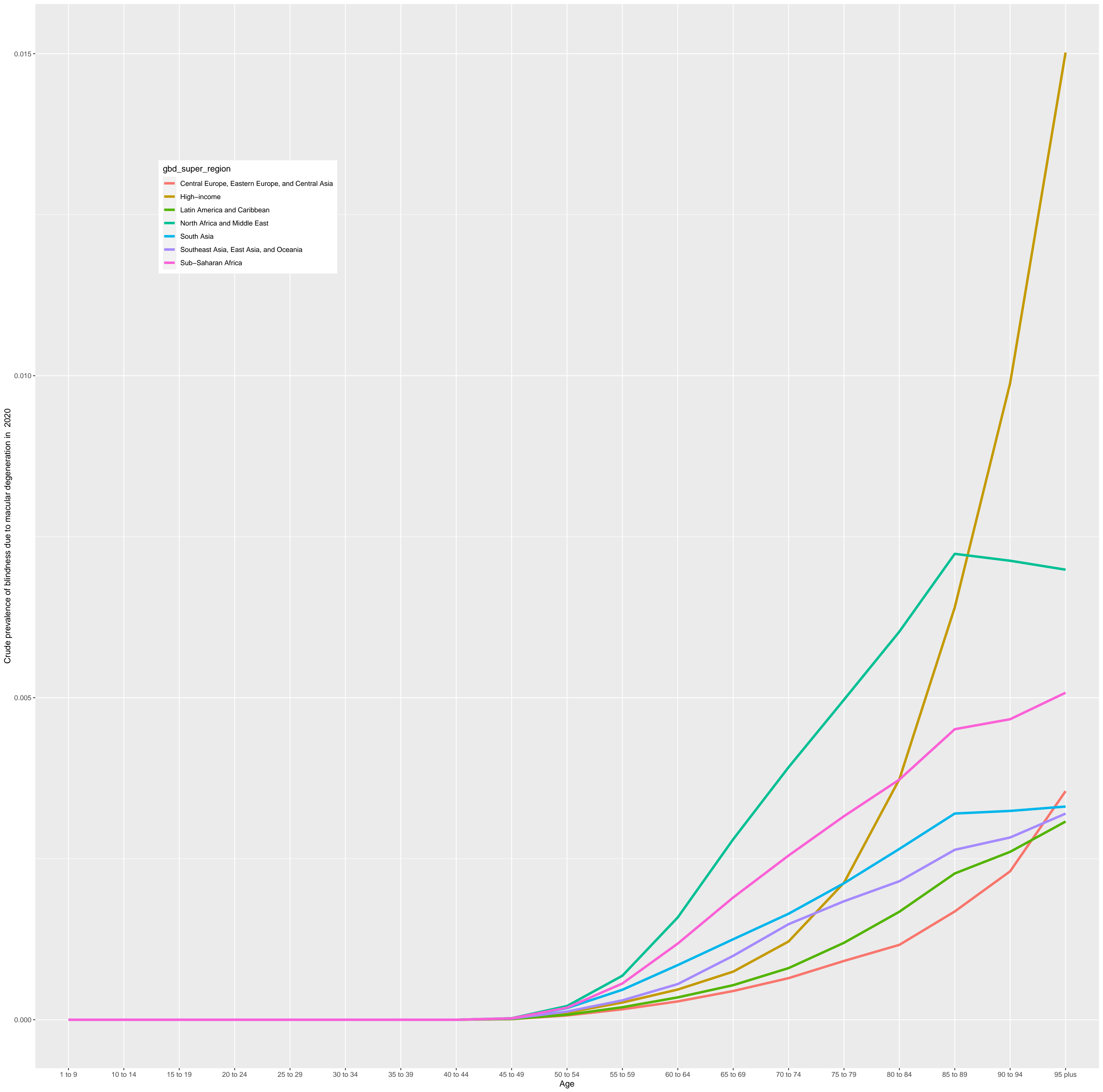

Supplement: Supplementary file 1 — Supplemental Figure 1 [file 41433_2024_3050_MOESM1_ESM.pdf]

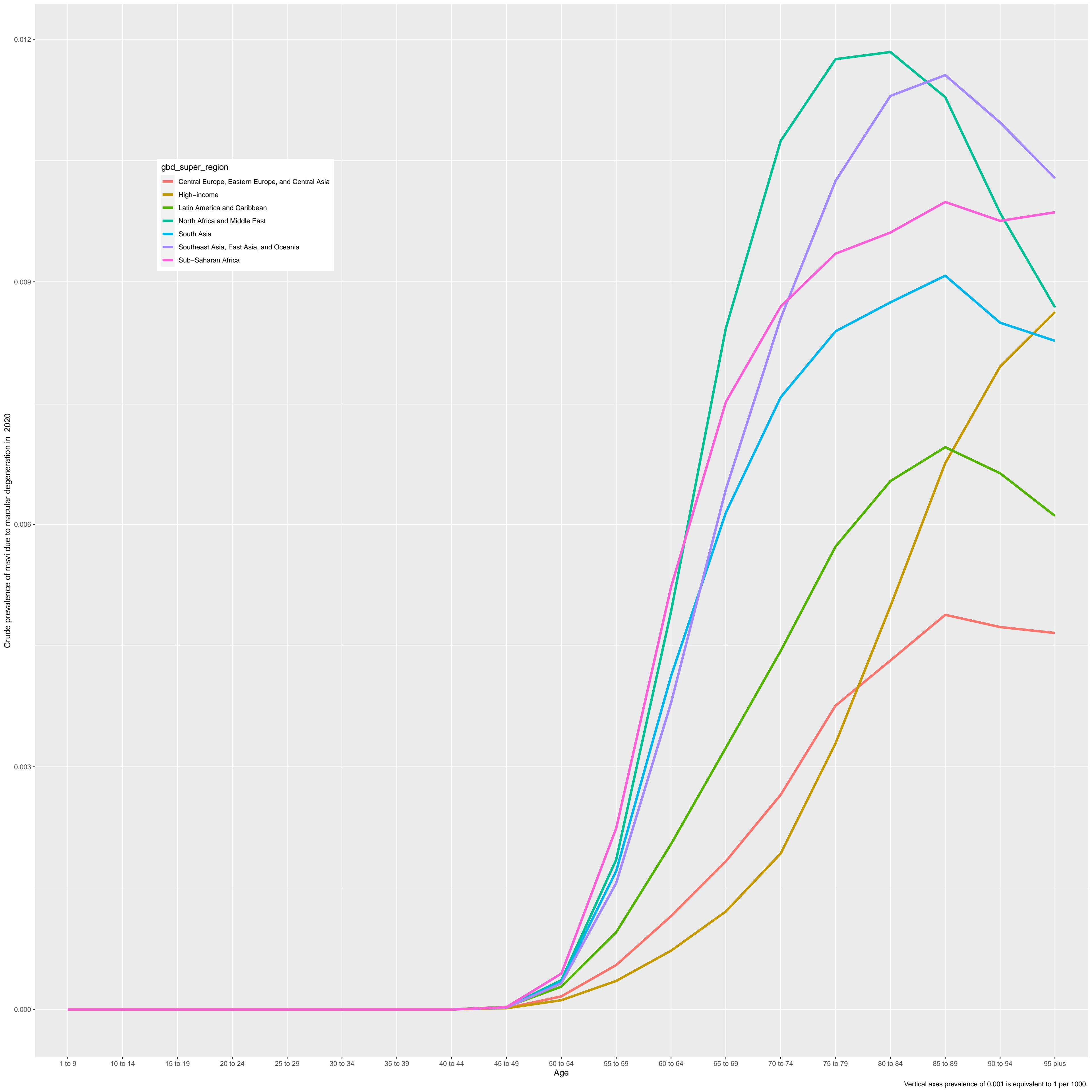

Supplement: Supplementary file 2 — Supplemental Figure 2 [file 41433_2024_3050_MOESM2_ESM.pdf]
