# Supplementary material for: Global estimates on the number of people blind or visually impaired by age-related macular degeneration: a meta-analysis from 2000 to 2020
Source: Eye (Lond). 2024 Jul 4;38(11):2070–82. doi: 10.1038/s41433-024-03050-z (PMC11269688; doi:10.1038/s41433-024-03050-z)
Supplement: Supplementary file 3 — Supplemental Table 1 [file 41433_2024_3050_MOESM3_ESM.pdf]

gbd_super_region	gbd_region	gbd_country	coverage	Reference
Central Europe, Eastern Europe, and Central Asia	Central Asia	Kyrgyzstan	Subnational	International Centre for Eye Health (ICEH). Kyrgyzstan - Batken, Jalal Abad, and Osh Rapid Assessment of Avoidable Blindness Survey 2017. Grootebroek, Netherlands: RAAB Repository, 2017.
Central Europe, Eastern Europe, and Central Asia	Central Asia	Mongolia	National	International Centre for Eye Health (ICEH). Mongolia Rapid Assessment of Avoidable Blindness Survey 2013. Grootebroek, Netherlands: RAAB Repository, 2014.
Central Europe, Eastern Europe, and Central Asia	Central Asia	Mongolia	Subnational	Baasanhu J, Johnson GJ, Burendei G, Minassian DC. Prevalence and causes of blindness and visual impairment in Mongolia: a survey of populations aged 40 years and older. Bull World Health Organ. 1994; 72(5): 771-6.
Central Europe, Eastern Europe, and Central Asia	Central Europe	Bulgaria	Subnational	Vassileva P, Gieser SC, Vitale S, Cholakova T, Katz J, West S. Blindness and visual impairment in western Bulgaria. Ophthalmic Epidemiol. 1996; 3(3): 143-9.
Central Europe, Eastern Europe, and Central Asia	Central Europe	Hungary	National	International Centre for Eye Health (ICEH). Hungary Rapid Assessment of Avoidable Blindness 2015. Grootebroek, Netherlands: RAAB Repository.
Central Europe, Eastern Europe, and Central Asia	Eastern Europe	Republic of Moldova	National	Zatic T1, Bendelic E2, Paduca A2, Rabiu M3, Corduneanu A2, Garaba A, Novac V, Curca C, Sorbala I, Chiaburu A, Verega F, Andronic V, Guzun I, Căpăţină O, Zamă-Mardari I. Rapid assessment of avoidable blindness and diabetic retinopathy in Republic of Moldova. *Br J Ophthalmol*. 2015; 99(6): 832-6.
Central Europe, Eastern Europe, and Central Asia	Eastern Europe	Russian Federation	Subnational	International Centre for Eye Health (ICEH). Russia - Samara District Rapid Assessment for Avoidable Blindness Survey 2008.
High-income	Australasia	Australia	Subnational	Centre for Vision Research, University of Sydney. Australia - Blue Mountains Eye Study 1992-1994.
High-income	Australasia	Australia	Subnational	Taylor HR, Livingston PM, Stanislavsky YL, McCarty CA. Visual impairment in Australia: distance visual acuity, near vision, and visual field findings of the Melbourne Visual Impairment Project. Am J Ophthalmol. 1997; 123(3): 328-37.

High-income	Australasia	Australia	Subnational	VanNewkirk MR, Weih L, McCarty CA, Taylor HR. Cause-specific prevalence of bilateral visual impairment in Victoria, Australia: the Visual Impairment Project. Ophthalmology. 2001; 108(5): 960-7.
High-income	High-income Asia Pacific	Singapore	Subnational	Saw S-M, Foster PJ, Gazzard G, Seah S. Causes of blindness, low vision, and questionnaire-assessed poor visual function in Singaporean Chinese adults: The Tanjong Pagar Survey. Ophthalmology. 2004; 111(6): 1161-8.
High-income	High-income Asia Pacific	Singapore	Subnational	Wong TY, Chong EW, Wong W-L, Rosman M, Aung T, Loo J-L, Shen S, Loon S-C, Tan DTH, Tai ES, Saw S-M. Prevalence and causes of low vision and blindness in an urban malay population: the Singapore Malay Eye Study. Arch Ophthalmol. 2008; 126(8): 1091-9.
High-income	High-income North America	United States of America	Subnational	Department of Ophthalmology and Visual Sciences, University of Wisconsin-Madison. United States - Wisconsin Beaver Dam Eye Study 1988-1990.
High-income	High-income North America	United States of America	Subnational	Johns Hopkins Bloomberg School of Public Health. United States - Arizona Proyecto VER Survey 2000.
High-income	Southern Latin America	Argentina	National	International Centre for Eye Health (ICEH). Argentina Rapid Assessment of Avoidable Blindness Survey 2013. Grootebroek, Netherlands: RAAB Repository, 2013.
High-income	Southern Latin America	Argentina	Subnational	International Centre for Eye Health (ICEH). Argentina - Buenos Aires Rapid Assessment of Avoidable Blindness 2003. Grootebroek, Netherlands: RAAB Repository.
High-income	Southern Latin America	Chile	Subnational	International Centre for Eye Health (ICEH). Chile - Biobio Rapid Assessment of Avoidable Blindness 2006. Grootebroek, Netherlands: RAAB Repository.
High-income	Southern Latin America	Uruguay	National	International Centre for Eye Health (ICEH). Uruguay Rapid Assessment of Avoidable Blindness 2011.
High-income	Western Europe	Denmark	Subnational	Buch H, Vinding T, La Cour M, Nielsen NV. The prevalence and causes of bilateral and unilateral blindness in an elderly urban Danish population. The Copenhagen City Eye Study. Acta Ophthalmol Scand. 2001; 79(5): 441-9.
High-income	Western Europe	Denmark	Subnational	Buch H, Vinding T, Nielsen NV. Prevalence and causes of visual impairment according to World Health

				Organization and United States criteria in an aged, urban Scandinavian population: the Copenhagen City Eye Study. Ophthalmology. 2001; 108(12): 2347-57.
High-income	Western Europe	Iceland	Subnational	Gunnlaugsdottir E, Arnarsson A, Jonasson F. Prevalence and causes of visual impairment and blindness in Icelanders aged 50 years and older: the Reykjavik Eye Study. Acta Ophthalmol. 2008; 86(7): 778-85.
High-income	Western Europe	Israel	Subnational	Thomson IM, Chumbley LC. Eye disease in the West Bank and Gaza Strip. Br J Ophthalmol. 1984; 68(8): 598-602.
High-income	Western Europe	Italy	Subnational	Cedrone C, Nucci C, Scuderi G, Ricci F, Cerulli A, Culasso F. Prevalence of blindness and low vision in an Italian population: a comparison with other European studies. Eye (Lond). 2006; 20(6): 661-7.
High-income	Western Europe	Netherlands	Subnational	Klaver CC, Wolfs RC, Vingerling JR, Hofman A, de Jong PT. Age-specific prevalence and causes of blindness and visual impairment in an older population: the Rotterdam Study. Arch Ophthalmol. 1998; 116(5): 653-8.
High-income	Western Europe	Spain	Subnational	Esteban JJN, Martínez MS, Navalón PG, Serrano OP, Patiño JRC, Purón MEC, Martínez-Vizcaíno V. Visual impairment and quality of life: gender differences in the elderly in Cuenca, Spain. *Qual Life Res*. 2008; 17(1): 37-45.
High-income	Western Europe	United Kingdom	National	Evans JR, Fletcher AE, Wormald RPL. Age-related macular degeneration causing visual impairment in people 75 years or older in Britain: an add-on study to the Medical Research Council Trial of Assessment and Management of Older People in the Community. Ophthalmology. 2004; 111(3): 513-7.
Latin America and Caribbean	Andean Latin America	Ecuador	National	International Centre for Eye Health (ICEH). Ecuador Rapid Assessment of Avoidable Blindness 2008-2009. Grootebroek, Netherlands: RAAB Repository.
Latin America and Caribbean	Andean Latin America	Peru	National	International Centre for Eye Health (ICEH). Peru Rapid Assessment of Avoidable Blindness 2011-2012. Grootebroek, Netherlands: RAAB Repository.
Latin America and Caribbean	Caribbean	Barbados	National	Ministry of Health (Barbados). Barbados Eye Study 1987-1992.

Latin America and Caribbean	Caribbean	Cuba	Subnational	Hernández Silva JR, Río Torres M, Padilla González CM. Resultados del RACSS en Ciudad de La Habana, Cuba, 2005. *Rev Cubana Oftalmol*. 2006; 19(1): 0-0.
Latin America and Caribbean	Caribbean	Dominican Republic	National	International Centre for Eye Health (ICEH). Dominican Republic Rapid Assessment for Avoidable Blindness Survey 2008.
Latin America and Caribbean	Caribbean	Suriname	National	Minderhoud J, Pawiroredjo JC, Themen HCI, Bueno de Mesquita-Voigt A-MT, Siban MR, Forster-Pawiroredjo CM, Limburg H, van Nispen RMA, Mans DRA, Moll AC. Blindness and Visual Impairment in the Republic of Suriname. *Ophthalmology*. 2015; 122(10): 2147–9.
Latin America and Caribbean	Central Latin America	Costa Rica	National	International Centre for Eye Health (ICEH). Costa Rica Rapid Assessment of Avoidable Blindness Survey 2015. Grootebroek, Netherlands: RAAB Repository, 2018.
Latin America and Caribbean	Central Latin America	El Salvador	National	International Centre for Eye Health (ICEH). El Salvador Rapid Assessment of Avoidable Blindness 2011.
Latin America and Caribbean	Central Latin America	Guatemala	National	International Centre for Eye Health (ICEH). Guatemala Rapid Assessment of Avoidable Blindness 2015. Grootebroek, Netherlands: RAAB Repository.
Latin America and Caribbean	Central Latin America	Guatemala	Subnational	International Centre for Eye Health (ICEH). Guatemala - Four Departments Rapid Assessment of Avoidable Blindness 2004. Grootebroek, Netherlands: RAAB Repository.
Latin America and Caribbean	Central Latin America	Honduras	National	International Centre for Eye Health (ICEH). Honduras Rapid Assessment of Avoidable Blindness 2013.
Latin America and Caribbean	Central Latin America	Mexico	Subnational	International Centre for Eye Health (ICEH), Tennent Institute of Ophthalmology, Vision Institute, Carlota Hospital (Nuevo LeÃ³n). Mexico - Chiapas Rapid Assessment of Avoidable Blindness and Diabetic Retinopathy 2010.
Latin America and Caribbean	Central Latin America	Mexico	Subnational	International Centre for Eye Health (ICEH). Mexico - Nuevo Leon Rapid Assessment of Avoidable Blindness 2005-2006. Grootebroek, Netherlands: RAAB Repository.
Latin America and Caribbean	Central Latin America	Mexico	Subnational	International Centre for Eye Health (ICEH). Mexico - Nuevo Leon Rapid Assessment of Avoidable Blindness

				Survey 2014. Grootebroek, Netherlands: RAAB Repository, 2014.
Latin America and Caribbean	Central Latin America	Mexico	Subnational	International Centre for Eye Health (ICEH). Mexico - Querétaro de Arteaga Rapid Assessment of Avoidable Blindness 2015-2016. Grootebroek, Netherlands: RAAB Repository.
Latin America and Caribbean	Central Latin America	Panama	National	International Centre for Eye Health (ICEH), Ophthalmology School, Specialized University of the Americas (Panama). Panama Rapid Assessment of Avoidable Blindness 2012-2014. Grootebroek, Netherlands: RAAB Repository.
Latin America and Caribbean	Central Latin America	Venezuela (Bolivarian Republic of)	National	International Centre for Eye Health (ICEH). Venezuela Rapid Assessment of Avoidable Blindness Survey 2004. Grootebroek, Netherlands: RAAB Repository.
Latin America and Caribbean	Tropical Latin America	Brazil	Subnational	Araújo Filho A, Salomão SR, Berezovsky A, Cinoto RW, Morales PHA, Santos FRG, Belfort R Jr. Prevalence of visual impairment, blindness, ocular disorders and cataract surgery outcomes in low-income elderly from a metropolitan region of São Paulo-Brazil. *Arq Bras Oftalmol*. 2008; 71(2): 246-53.
Latin America and Caribbean	Tropical Latin America	Brazil	Subnational	International Centre for Eye Health (ICEH). Brazil - Campinas Rapid Assessment of Avoidable Blindness 2004. Grootebroek, Netherlands: RAAB Repository.
Latin America and Caribbean	Tropical Latin America	Brazil	Subnational	Schellini SA, Durkin SR, Hoyama E, Hirai F, Cordeiro R, Casson RJ, Selva D, Padovani CR. Prevalence and causes of visual impairment in a Brazilian population: the Botucatu Eye Study. BMC Ophthalmol. 2009; 9: 8.
Latin America and Caribbean	Tropical Latin America	Paraguay	National	International Centre for Eye Health (ICEH). Paraguay Rapid Assessment of Avoidable Blindness 2011.
North Africa and Middle East	North Africa and Middle East	Afghanistan	Subnational	Comprehensive Health and Education Forum International (CHEF) (Pakistan), International Centre for Eye Health (ICEH). Afghanistan Rapid Assessment of Avoidable Blindness 2010.
North Africa and Middle East	North Africa and Middle East	Iran (Islamic Republic of)	Subnational	International Centre for Eye Health (ICEH). Iran - Kordestan Rapid Assessment of Avoidable Blindness 2014. Grootebroek, Netherlands: RAAB Repository.

North Africa and Middle East	North Africa and Middle East	Iran (Islamic Republic of)	Subnational	International Centre for Eye Health (ICEH). Iran - Varamin Rapid Assessment of Avoidable Blindness 2009. Grootebroek, Netherlands: RAAB Repository.
North Africa and Middle East	North Africa and Middle East	Jordan	National	Rabiu MM, Al Bdour MD, Abu Ameerh MA, Jadoon MZ. Prevalence of blindness and diabetic retinopathy in northern Jordan. Eur J Ophthalmol. 2015; 25(4): 320-7.
North Africa and Middle East	North Africa and Middle East	Lebanon	National	Mansour AM, Kassak K, Chaya M, Hourani T, Sibai A, Alameddine MN. National survey of blindness and low vision in Lebanon. Br J Ophthalmol. 1997; 81(10): 905-6.
North Africa and Middle East	North Africa and Middle East	Libya	National	Rabiu MM, Jenf M, Fituri S, Choudhury A, Agbabiaka I, Mousa A. Prevalence and causes of visual impairment and blindness, cataract surgical coverage and outcomes of cataract surgery in Libya. Ophthalmic Epidemiol. 2013; 20(1): 26-32.
North Africa and Middle East	North Africa and Middle East	Morocco	National	World Health Organization (WHO). Prevention of blindness (PBL). Prevalence and causes of blindness and low vision.Wkly Epidemiol Rec. 1994: 69(18): 129-31.
North Africa and Middle East	North Africa and Middle East	Oman	National	Khandekar R, Mohammed AJ, Negrel AD, Riyami AA. The prevalence and causes of blindness in the Sultanate of Oman: the Oman Eye Study (OES). Br J Ophthalmol. 2002; 86(9): 957-62.
North Africa and Middle East	North Africa and Middle East	Oman	National	Khandekar R, Mohammed AJ, Raisi AA. Prevalence and causes of blindness and low vision: before and five years after "VISION 2020" initiatives in Oman: a review. Ophthalmic Epidemiol. 2007; 14(1): 9-15.
North Africa and Middle East	North Africa and Middle East	Palestine	National	Chiang F, Kuper H, Lindfield R, Keenan T, Seyam N, Magauran D, Khalilia N, Batta H, Abdeen Z, Sargent N. Rapid assessment of avoidable blindness in the Occupied Palestinian Territories. PLoS One. 2010; 5(7): e11854.
North Africa and Middle East	North Africa and Middle East	Palestine	National	International Centre for Eye Health (ICEH). Palestine Rapid Assessment of Avoidable Blindness 2008. Grootebroek, Netherlands: RAAB Repository.
North Africa and Middle East	North Africa and Middle East	Qatar	National	Al Gamra H, Al Mansouri F, Khandekar R, Elshafei M, Al Qahtani O, Singh R, Hashim SP, Mujahed A, Makled A, Pai A. Prevalence and causes of blindness, low vision and status of cataract in 50 years and older citizen of Qatar –

				a community based survey. *Ophthalmic Epidemiol*. 2010; 17(5): 292-300.
North Africa and Middle East	North Africa and Middle East	Sudan	Subnational	International Centre for Eye Health (ICEH). Sudan - North Kordofan Rapid Assessment of Avoidable Blindness 2010. Grootebroek, Netherlands: RAAB Repository.
North Africa and Middle East	North Africa and Middle East	Sudan	Subnational	International Centre for Eye Health (ICEH). Sudan - Northern Rapid Assessment of Avoidable Blindness 2009-2010. Grootebroek, Netherlands: RAAB Repository.
North Africa and Middle East	North Africa and Middle East	Sudan	Subnational	International Centre for Eye Health (ICEH). Sudan - Sennar Rapid Assessment of Avoidable Blindness 2010. Grootebroek, Netherlands: RAAB Repository.
North Africa and Middle East	North Africa and Middle East	Sudan	Subnational	International Centre for Eye Health (ICEH). Sudan - White Nile Rapid Assessment of Avoidable Blindness Survey 2010. Grootebroek, Netherlands: RAAB Repository, 2010.
North Africa and Middle East	North Africa and Middle East	Yemen	Subnational	Al-Khatib TK, Ahmed AA, Hameed AS. Rapid assessment of avoidable blindness in amran and lahj governorates of Yemen. *Sudan J Ophthalmol*. 2017; 5(1): 9–16.
North Africa and Middle East	North Africa and Middle East	Yemen	Subnational	International Centre for Eye Health (ICEH). Yemen - Amran Rapid Assessment of Avoidable Blindness 2009.
South Asia	South Asia	Bangladesh	National	International Centre for Eye Health (ICEH), National Institute of Ophthalmology (Bangladesh). Bangladesh National Blindness and Low Vision Prevalence Survey 1999-2000.
South Asia	South Asia	Bangladesh	Subnational	CSF Global (Bangladesh), International Centre for Eye Health (ICEH). Bangladesh - Barisal Rapid Assessment of Avoidable Blindness 2013.
South Asia	South Asia	Bangladesh	Subnational	CSF Global (Bangladesh), International Centre for Eye Health (ICEH). Bangladesh - Brahmanbaria and Satkhira Districts Rapid Assessment of Avoidable Blindness 2012.
South Asia	South Asia	Bangladesh	Subnational	CSF Global (Bangladesh), International Centre for Eye Health (ICEH). Bangladesh - Gazipur, Kishoreganj, and Cox's Bazar Districts Rapid Assessment of Avoidable Blindness 2010. Grootebroek, Netherlands: RAAB Repository.

South Asia	South Asia	Bangladesh	Subnational	CSF Global (Bangladesh), International Centre for Eye Health (ICEH). Bangladesh - Narail and Jamalpur Districts Rapid Assessment of Avoidable Blindness 2010.
South Asia	South Asia	Bangladesh	Subnational	CSF Global (Bangladesh), International Centre for Eye Health (ICEH). Bangladesh - Satkhira District Rapid Assessment of Avoidable Blindness 2005. Grootebroek, Netherlands: RAAB Repository.
South Asia	South Asia	Bangladesh	Subnational	CSF Global (Bangladesh), International Centre for Eye Health (ICEH). Bangladesh - Tangail Rapid Assessment of Avoidable Blindness 2011. Grootebroek, Netherlands: RAAB Repository.
South Asia	South Asia	Bangladesh	Subnational	International Centre for Eye Health (ICEH). Bangladesh - Kushtia Rapid Assessment of Avoidable Blindness Survey 2011. Grootebroek, Netherlands: RAAB Repository, 2011.
South Asia	South Asia	Bhutan	National	International Centre for Eye Health (ICEH). Bhutan Rapid Assessment of Avoidable Blindness Survey 2009. Grootebroek, Netherlands: RAAB Repository, 2009.
South Asia	South Asia	Bhutan	National	Lepcha NT, Chettri CK, Getshen K, Rai BB, Ramaswamy SB, Saibaba S, Nirmalan PK, Demarchis EH, Tabin G, Morley M, Morley K. Rapid assessment of avoidable blindness in Bhutan. Ophthalmic Epidemiol. 2013; 20(4): 212-9.
South Asia	South Asia	India	Subnational	All India Institute of Medical Sciences, New Delhi (AIIMS). India Multi-centric Collaborative Study on the Impact of Global Warming and Ultra Violet Radiation Exposure on Ocular Health in India 2013-2015.
South Asia	South Asia	India	Subnational	Bettadapura GS, Donthi K, Datti NP, Ranganath BG, Ramaswamy SB, Jayaram TS. Assessment of avoidable blindness using the rapid assessment of avoidable blindness methodology. N Am J Med Sci. 2012; 4(9): 389-93.
South Asia	South Asia	India	Subnational	Dandona L, Dandona R, Srinivas M, Giridhar P, Vilas K, Prasad MN, John RK, McCarty CA, Rao GN. Blindness in the Indian state of Andhra Pradesh. Invest Ophthalmol Vis Sci. 2001; 42(5): 908-16.

South Asia	South Asia	India	Subnational	International Centre for Eye Health (ICEH). India - Chitrakoot Rapid Assessment of Avoidable Blindness 2008. Grootebroek, Netherlands: RAAB Repository.
South Asia	South Asia	India	Subnational	International Centre for Eye Health (ICEH). India - Surat Rapid Assessment of Avoidable Blindness 2011.
South Asia	South Asia	India	Subnational	Murthy GVS, Gupta SK, Bachani D, Jose R, John N, National Programme for Control of Blindness (India). Current estimates of blindness in India [Unpublished data]. Br J Ophthalmol. 2005; 89(3): 257-60.
South Asia	South Asia	India	Subnational	Nangia V, Jonas JB, Kulkarni M, Matin A. Prevalence of age-related macular degeneration in rural central India: the Central India Eye and Medical Study. Retina. 2011; 31(6): 1179-85.
South Asia	South Asia	India	Subnational	Neena J, Rachel J, Praveen V, Murthy GVS. Rapid Assessment of Avoidable Blindness in India [Unpublished data]. PLoS One. 2008; 3(8): e2867.
South Asia	South Asia	India	Subnational	Nirmalan PK, Katz J, Robin AL, Tielsch JM, Namperumalsamy P, Kim R, Narendran V, Ramakrishnan R, Krishnadas R, Thulasiraj RD, Suan E. Prevalence of vitreoretinal disorders in a rural population of southern India: the Aravind Comprehensive Eye Study. Arch Ophthalmol. 2004; 122(4): 581-6.
South Asia	South Asia	India	Subnational	Nirmalan PK, Vijayalakshmi P, Sheeladevi S, Kothari MB, Sundaresan K, Rahmathullah L. The Kariapatti pediatric eye evaluation project: baseline ophthalmic data of children aged 15 years or younger in Southern India. Am J Ophthalmol. 2003; 136(4): 703-9.
South Asia	South Asia	India	Subnational	Thulasiraj RD, Nirmalan PK, Ramakrishnan R, Krishnadas R, Manimekalai TK, Baburajan NP, Katz J, Tielsch JM, Robin AL. Blindness and vision impairment in a rural south Indian population: the Aravind Comprehensive Eye Survey. Ophthalmology. 2003; 110(8): 1491-8.
South Asia	South Asia	India	Subnational	Vijaya L, George R, Arvind H, Baskaran M, Raju P, Ramesh SV, Paul PG, Kumaramanickavel G, McCarty C. Prevalence and causes of blindness in the rural population of the

				Chennai Glaucoma Study. Br J Ophthalmol. 2006; 90(4): 407-10.
South Asia	South Asia	India	Subnational	Vijaya L, George R, Asokan R, Velumuri L, Ramesh SV. Prevalence and causes of low vision and blindness in an urban population: The Chennai Glaucoma Study. Indian J Ophthalmol. 2014.
South Asia	South Asia	Nepal	Subnational	Dulal S, Sapkota YD. Prevalence of blindness and visual impairment and its causes among people aged 50 years and above in Karnali Zone, Nepal. Nepal J Ophthalmol. 2012; 4(2).
South Asia	South Asia	Nepal	Subnational	International Centre for Eye Health (ICEH), Tilganga Institute of Ophthalmology. Nepal - Bagmati Zone Rapid Assessment for Avoidable Blindness 2008.
South Asia	South Asia	Nepal	Subnational	International Centre for Eye Health (ICEH). Nepal - Bheri Zone Rapid Assessment of Avoidable Blindness 2009.
South Asia	South Asia	Nepal	Subnational	International Centre for Eye Health (ICEH). Nepal - Dhaulagiri Zone Rapid Assessment of Avoidable Blindness 2010.
South Asia	South Asia	Nepal	Subnational	International Centre for Eye Health (ICEH). Nepal - Janakpur Rapid Assessment for Avoidable Blindness 2008. Grootebroek, Netherlands: RAAB Repository.
South Asia	South Asia	Nepal	Subnational	International Centre for Eye Health (ICEH). Nepal - Karnali Zone Rapid Assessment of Avoidable Blindness 2008.
South Asia	South Asia	Nepal	Subnational	International Centre for Eye Health (ICEH). Nepal - Koshi Zone Rapid Assessment of Avoidable Blindness 2008-2009.
South Asia	South Asia	Nepal	Subnational	International Centre for Eye Health (ICEH). Nepal - Mechi Zone Rapid Assessment of Avoidable Blindness 2009.
South Asia	South Asia	Nepal	Subnational	International Centre for Eye Health (ICEH). Nepal - Narayani Rapid Assessment of Avoidable Blindness 2015-2016. Grootebroek, Netherlands: RAAB Repository.
South Asia	South Asia	Nepal	Subnational	International Centre for Eye Health (ICEH). Nepal - Rapti Zone Rapid Assessment of Avoidable Blindness 2010.
South Asia	South Asia	Nepal	Subnational	International Centre for Eye Health (ICEH). Nepal - Sagarmatha Zone Rapid Assessment of Avoidable Blindness 2008-2009.

South Asia	South Asia	Nepal	Subnational	International Centre for Eye Health (ICEH). Nepal - Seti and Mahakali Zones Rapid Assessment of Avoidable Blindness 2008.
South Asia	South Asia	Nepal	Subnational	Thapa R, Bajimaya S, Paudyal G, Khanal S, Tan S, Thapa SS, van Rens GHMB. Prevalence and causes of low vision and blindness in an elderly population in Nepal: the Bhaktapur retina study. BMC Ophthalmol. 2018; 18(1): 42.
South Asia	South Asia	Pakistan	National	Pakistan National Survey on Blindness and Low Vision 2002-2004. [Unpublished].
South Asia	South Asia	Pakistan	Subnational	Ahmad K, Khan MD, Qureshi MB, Munami S, Shah RA, Rasheed H, Jamali B, Baluch A, Khan MA. Prevalence and causes of blindness and low vision in a rural setting in Pakistan. Ophthalmic Epidemiol. 2005; 12(1): 19-23.
South Asia	South Asia	Pakistan	Subnational	International Centre for Eye Health (ICEH). Pakistan - Chakwal Rapid Assessment of Avoidable Blindness 2000.
South Asia	South Asia	Pakistan	Subnational	International Centre for Eye Health (ICEH). Pakistan - Peshawar Rapid Assessment of Avoidable Blindness 2013. Grootebroek, Netherlands: RAAB Repository.
Southeast Asia, East Asia, and Oceania	East Asia	China	Subnational	Chen X, Zhou D, Shen J, Wu Y, Sun Q, Dong J, Yu J. Prevalence and Causes of Visual Impairment in Adults in Binhu District, Wuxi, China. Med Sci Monit. 2018; 24: 317-323.
Southeast Asia, East Asia, and Oceania	East Asia	China	Subnational	Cheng F, Shan L, Song W, Fan P, Yuan H. Distance- and near-visual impairment in rural Chinese adults in Kailu, Inner Mongolia. Acta Ophthalmol. 2016; 94(4): 407-13.
Southeast Asia, East Asia, and Oceania	East Asia	China	Subnational	International Centre for Eye Health (ICEH). China - Gao'an Rapid Assessment of Avoidable Blindness 2007. Grootebroek, Netherlands: RAAB Repository.
Southeast Asia, East Asia, and Oceania	East Asia	China	Subnational	International Centre for Eye Health (ICEH). China - Inner Mongolia Shangdu Rapid Assessment of Avoidable Blindness 2010.
Southeast Asia, East Asia, and Oceania	East Asia	China	Subnational	International Centre for Eye Health (ICEH). China - Inner Mongolia Tuoketuo Rapid Assessment of Avoidable Blindness 2010.

Southeast Asia, East Asia, and Oceania	East Asia	China	Subnational	International Centre for Eye Health (ICEH). China - Jianchuan Rapid Assessment of Avoidable Blindness 2012. Grootebroek, Netherlands: RAAB Repository.
Southeast Asia, East Asia, and Oceania	East Asia	China	Subnational	International Centre for Eye Health (ICEH). China - Kunming Rapid Assessment of Avoidable Blindness 2006. Grootebroek, Netherlands: RAAB Repository.
Southeast Asia, East Asia, and Oceania	East Asia	China	Subnational	International Centre for Eye Health (ICEH). China - Lancang Rapid Assessment of Avoidable Blindness 2012. Grootebroek, Netherlands: RAAB Repository.
Southeast Asia, East Asia, and Oceania	East Asia	China	Subnational	International Centre for Eye Health (ICEH). China - Sichuan Dechang Rapid Assessment of Avoidable Blindness 2011.
Southeast Asia, East Asia, and Oceania	East Asia	China	Subnational	International Centre for Eye Health (ICEH). China - Sichuan Garzé Rapid Assessment of Avoidable Blindness Survey 2017. Grootebroek, Netherlands: RAAB Repository.[Unpublished].
Southeast Asia, East Asia, and Oceania	East Asia	China	Subnational	International Centre for Eye Health (ICEH). China - Sichuan Mianning Rapid Assessment of Avoidable Blindness 2011.
Southeast Asia, East Asia, and Oceania	East Asia	China	Subnational	International Centre for Eye Health (ICEH). China - Wanzai Rapid Assessment of Avoidable Blindness 2007. Grootebroek, Netherlands: RAAB Repository.
Southeast Asia, East Asia, and Oceania	East Asia	China	Subnational	International Centre for Eye Health (ICEH). China - Xingan Rapid Assessment of Avoidable Blindness 2007. Grootebroek, Netherlands: RAAB Repository.
Southeast Asia, East Asia, and Oceania	East Asia	China	Subnational	International Centre for Eye Health (ICEH). China - Xinjiang Rapid Assessment of Avoidable Blindness 2015. Grootebroek, Netherlands: RAAB Repository.
Southeast Asia, East Asia, and Oceania	East Asia	China	Subnational	International Centre for Eye Health (ICEH). China - Yunnan Luliang Rapid Assessment of Avoidable Blindness 2008. Grootebroek, Netherlands: RAAB Repository, 2008.
Southeast Asia, East Asia, and Oceania	East Asia	China	Subnational	Li EY, Liu Y, Zhan X, Liang YB, Zhang X, Zheng C, Jhanji V, Xu P, Chang DF, Lam DS. Prevalence of blindness and outcomes of cataract surgery in Hainan Province in South China. Ophthalmology. 2013; 120(11.0): 2176-83.

Southeast Asia, East Asia, and Oceania	East Asia	China	Subnational	Li J, Zhong H, Cai N, Luo T, Li J, Su X, Li X, Qiu X, Yang Y, Yuan Y, Yu M. The prevalence and causes of visual impairment in an elderly Chinese Bai ethnic rural population: the Yunnan minority eye study. Invest Ophthalmol Vis Sci. 2012; 53(8): 4498-504.
Southeast Asia, East Asia, and Oceania	East Asia	China	Subnational	Li L, Guan H, Xun P, Zhou J, Gu H. Prevalence and causes of visual impairment among the elderly in Nantong, China. Eye (Lond). 2008; 22(8): 1069-75.
Southeast Asia, East Asia, and Oceania	East Asia	China	Subnational	Li Y, Bi HS, Wang LH, Wang T, Yang SY, Liu LP, Zhou CC. Causes of moderate to severe visual impairment and blindness in population aged 50 years or more in rural Shandong province. Chin J Ophthalmol. 2013; 49(2): 144-50.
Southeast Asia, East Asia, and Oceania	East Asia	China	Subnational	Li Z, Cui H, Liu P, Zhang L, Yang H, Zhang L. Prevalence and causes of blindness and visual impairment among the elderly in rural southern Harbin, China. Ophthalmic Epidemiol. 2008; 15(5): 334-8.
Southeast Asia, East Asia, and Oceania	East Asia	China	Subnational	Tang Y, Wang X, Wang J, Huang W, Gao Y, Luo Y, Lu Y. Prevalence and Causes of Visual Impairment in a Chinese Adult Population: The Taizhou Eye Study. Ophthalmology. 2015; 122(7): 1480-8.
Southeast Asia, East Asia, and Oceania	East Asia	China	Subnational	Wang H, Zhang Y, Li Z, Wang T, Liu P. Prevalence and causes of corneal blindness. Clin Experiment Ophthalmol. 2013.
Southeast Asia, East Asia, and Oceania	East Asia	China	Subnational	Wang L, Huang W, He M, Zheng Y, Huang S, Liu B, Jin L, Congdon NG, He M. Causes and five-year incidence of blindness and visual impairment in urban Southern China: the Liwan Eye Study. Invest Ophthalmol Vis Sci. 2013; 54(6): 4117-21.
Southeast Asia, East Asia, and Oceania	East Asia	China	Subnational	Wu M, Yip JLY, Kuper H. Rapid assessment of avoidable blindness in Kunming, China. Ophthalmology. 2008; 115(6): 969-74.
Southeast Asia, East Asia, and Oceania	East Asia	China	Subnational	Xu J, Xu L, Du KF, Shao L, Chen CX, Zhou JQ, Wang YX, You QS, Jonas JB, Wei WB. Subfoveal choroidal thickness in diabetes and diabetic retinopathy [Unpublished data]. Ophthalmology. 2013 Oct;120(10):2023-8.

Southeast Asia, East Asia, and Oceania	East Asia	China	Subnational	Zhang G, Li Y, Teng X, Wu Q, Gong H, Ren F, Guo Y, Liu L, Zhang H. Prevalence and causes of low vision and blindness in Baotou: A cross-sectional study. Medicine (Baltimore). 2016; 95(37): e4905.
Southeast Asia, East Asia, and Oceania	East Asia	Taiwan (Province of China)	Subnational	Chen S-J, Cheng C-Y, Li A-F, Peng K-L, Chou P, Chiou S-H, Hsu W-M. Prevalence and associated risk factors of myopic maculopathy in elderly Chinese: the Shihpai eye study. Invest Ophthalmol Vis Sci. 2012; 53(8): 4868-73.
Southeast Asia, East Asia, and Oceania	East Asia	Taiwan (Province of China)	Subnational	Hsu WM, Cheng CY, Liu JH, Tsai SY, Chou P. Prevalence and causes of visual impairment in an elderly Chinese population in Taiwan: the Shihpai Eye Study. Ophthalmology. 2004; 111(1): 62-9.
Southeast Asia, East Asia, and Oceania	Oceania	Papua New Guinea	Subnational	Garap JN, Sheeladevi S, Shamanna BR, Nirmalan PK, Brian G, Williams C. Blindness and vision impairment in the elderly of Papua New Guinea. Clin Experiment Ophthalmol. 2006; 34(4): 335-41.
Southeast Asia, East Asia, and Oceania	Oceania	Vanuatu	National	Newland HS, Harris MF, Walland M, McKnight D, Galbraith JE, Iwasaki W, Momomura K. Epidemiology of blindness and visual impairment in Vanuatu. Bull World Health Organ. 1992; 70(3): 369-72.
Southeast Asia, East Asia, and Oceania	Southeast Asia	Cambodia	National	International Centre for Eye Health (ICEH). Cambodia Rapid Assessment for Avoidable Blindness 2007.
Southeast Asia, East Asia, and Oceania	Southeast Asia	Cambodia	Subnational	International Centre for Eye Health (ICEH), Subcommittee for the Prevention of Blindness (Cambodia). Cambodia - Battambang Rapid Assessment of Cataract Surgical Services 2002.
Southeast Asia, East Asia, and Oceania	Southeast Asia	Cambodia	Subnational	International Centre for Eye Health (ICEH), Subcommittee for the Prevention of Blindness (Cambodia). Cambodia - Kampong Cham Rapid Assessment of Cataract Surgical Services 2002.
Southeast Asia, East Asia, and Oceania	Southeast Asia	Cambodia	Subnational	International Centre for Eye Health (ICEH), Subcommittee for the Prevention of Blindness (Cambodia). Cambodia - Kampot Rapid Assessment of Cataract Surgical Services 2002.

Southeast Asia, East Asia, and Oceania	Southeast Asia	Cambodia	Subnational	International Centre for Eye Health (ICEH). Cambodia - Takeo Rapid Assessment for Avoidable Blindness 2011-2012. Grootebroek, Netherlands: RAAB Repository.
Southeast Asia, East Asia, and Oceania	Southeast Asia	Indonesia	Subnational	International Centre for Eye Health (ICEH). Indonesia - East Kalimantan Rapid Assessment of Avoidable Blindness 2006.
Southeast Asia, East Asia, and Oceania	Southeast Asia	Indonesia	Subnational	Saw S-M, Husain R, Gazzard GM, Koh D, Widjaja D, Tan DTH. Causes of low vision and blindness in rural Indonesia. Br J Ophthalmol. 2003; 87(9): 1075-8.
Southeast Asia, East Asia, and Oceania	Southeast Asia	Lao People's Democratic Republic	National	International Centre for Eye Health (ICEH). Laos Rapid Assessment of Avoidable Blindness Survey 2007. Grootebroek, Netherlands: RAAB Repository, 2017.
Southeast Asia, East Asia, and Oceania	Southeast Asia	Malaysia	National	Zainal M, Ismail SM, Ropilah AR, Elias H, Arumugam G, Alias D, Fathilah J, Lim TO, Ding LM, Goh PP. Prevalence of blindness and low vision in Malaysian population: results from the National Eye Survey 1996. Br J Ophthalmol. 2002; 86(9): 951-6.
Southeast Asia, East Asia, and Oceania	Southeast Asia	Malaysia	Subnational	International Centre for Eye Health (ICEH). Malaysia - Kelantan, Terengganu, and Pahang Rapid Assessment of Avoidable Blindness Survey 2014. Grootebroek, Netherlands: RAAB Repository, 2014.
Southeast Asia, East Asia, and Oceania	Southeast Asia	Malaysia	Subnational	International Centre for Eye Health (ICEH). Malaysia - Sabah Rapid Assessment of Avoidable Blindness Survey 2014. Grootebroek, Netherlands: RAAB Repository, 2014.
Southeast Asia, East Asia, and Oceania	Southeast Asia	Malaysia	Subnational	International Centre for Eye Health (ICEH). Malaysia - Sarawak Rapid Assessment of Avoidable Blindness Survey 2014. Grootebroek, Netherlands: RAAB Repository, 2014.
Southeast Asia, East Asia, and Oceania	Southeast Asia	Malaysia	Subnational	Zainal M, Masran L, Ropilah AR. Blindness and visual impairment amongst rural Malays in Kuala Selangor, Selangor. Med J Malaysia. 1998; 53(1): 46-50.
Southeast Asia, East Asia, and Oceania	Southeast Asia	Maldives	National	International Centre for Eye Health (ICEH). Maldives Rapid Assessment of Avoidable Blindness 2016. Grootebroek, Netherlands: RAAB Repository.
Southeast Asia, East Asia, and Oceania	Southeast Asia	Myanmar	Subnational	Casson RJ, Newland HS, Muecke J, McGovern S, Durkin S, Sullivan T, Oo TZ, Aung TH, Shein WK, Selva D, Aung T. Prevalence and causes of visual impairment in rural

				Myanmar: the Meiktila Eye Study. Ophthalmology. 2007; 114(12): 2302-8.
Southeast Asia, East Asia, and Oceania	Southeast Asia	Myanmar	Subnational	International Centre for Eye Health (ICEH). Myanmar - Taungoo District Rapid Assessment of Cataract Surgical Services Report 2001.
Southeast Asia, East Asia, and Oceania	Southeast Asia	Myanmar	Subnational	Limburg H, Maung N, Khin AS, Mynt TW. Study report on rapid assessment of cataract surgical services in Hinthada Township, Union of Myanmar. Naypyidaw, Myanmar: Ministry of Health, Department of Health, Trachoma Control and Prevention of Blindness Programme.
Southeast Asia, East Asia, and Oceania	Southeast Asia	Myanmar	Subnational	Limburg H, Maung N, Khin AS, Mynt TW. Study report on rapid assessment of cataract surgical services in Monywa District, Union of Myanmar. Naypyidaw, Myanmar: Ministry of Health, Department of Health, Trachoma Control and Prevention of Blindness Programme.
Southeast Asia, East Asia, and Oceania	Southeast Asia	Myanmar	Subnational	Limburg H, Maung N, Khin AS, Mynt TW. Study report on rapid assessment of cataract surgical services in Shwebo District, Union of Myanmar. Naypyidaw, Myanmar: Ministry of Health, Department of Health, Trachoma Control and Prevention of Blindness Programme.
Southeast Asia, East Asia, and Oceania	Southeast Asia	Philippines	Subnational	International Centre for Eye Health (ICEH). Philippines - Antique Rapid Assessment of Avoidable Blindness 2006. Grootebroek, Netherlands: RAAB Repository.
Southeast Asia, East Asia, and Oceania	Southeast Asia	Philippines	Subnational	International Centre for Eye Health (ICEH). Philippines - Negros Island Rapid Assessment of Avoidable Blindness 2005. Grootebroek, Netherlands: RAAB Repository.
Southeast Asia, East Asia, and Oceania	Southeast Asia	Thailand	National	Isipradit S, Sirimaharaj M, Charukamnoetkanok P, Thonginnetra O, Wongsawad W, Sathornsumetee B, Somboonthanakij S, Soomsawasdi P, Jitawatanarat U, Taweebanjongsin W, Arayangkoon E, Arame P, Kobkoonthon C, Pangputhipong P. The first rapid assessment of avoidable blindness (RAAB) in Thailand. PLoS One. 2014; 9(12): e114245.
Southeast Asia, East Asia, and Oceania	Southeast Asia	Thailand	National	Thailand National Survey of Blindness and Low Vision 1994. [Unpublished].

Southeast Asia, East Asia, and Oceania	Southeast Asia	Thailand	Subnational	Bourne RRA, Sukudom P, Foster PJ, Tantisevi V, Jitapunkul S, Lee PS, Johnson GJ, Rojanapongpun P. Prevalence of glaucoma in Thailand: a population based survey in Rom Klao District, Bangkok. Br J Ophthalmol. 2003; 87(9): 1069-74.
Southeast Asia, East Asia, and Oceania	Southeast Asia	Thailand	Subnational	Singalavanija A, Metheetrairut A, Ruangvaravate N, Tuchinda R, Wanumkarng N. Ocular diseases and blindness in elderly Thais. J Med Assoc Thai. 2001; 84(10): 1383-8.
Southeast Asia, East Asia, and Oceania	Southeast Asia	Timor-Leste	National	International Centre for Eye Health (ICEH). Timor Leste Rapid Assessment of Avoidable Blindness 2016. Grootebroek, Netherlands: RAAB Repository.
Southeast Asia, East Asia, and Oceania	Southeast Asia	Viet Nam	Subnational	International Centre for Eye Health (ICEH). Vietnam - Ba Ria-Vung Tau Rapid Assessment of Avoidable Blindness 2007. Grootebroek, Netherlands: RAAB Repository.
Southeast Asia, East Asia, and Oceania	Southeast Asia	Viet Nam	Subnational	International Centre for Eye Health (ICEH). Vietnam - Bac Ninh Rapid Assessment of Avoidable Blindness 2007. Grootebroek, Netherlands: RAAB Repository.
Southeast Asia, East Asia, and Oceania	Southeast Asia	Viet Nam	Subnational	International Centre for Eye Health (ICEH). Vietnam - Bac Ninh Rapid Assessment of Avoidable Blindness Survey 2015. Grootebroek, Netherlands: RAAB Repository.
Southeast Asia, East Asia, and Oceania	Southeast Asia	Viet Nam	Subnational	International Centre for Eye Health (ICEH). Vietnam - Binh Dinh Rapid Assessment of Avoidable Blindness 2007. Grootebroek, Netherlands: RAAB Repository.
Southeast Asia, East Asia, and Oceania	Southeast Asia	Viet Nam	Subnational	International Centre for Eye Health (ICEH). Vietnam - Binh Dinh Rapid Assessment of Cataract Surgical Services 2001. Grootebroek, Netherlands: RAAB Repository.
Southeast Asia, East Asia, and Oceania	Southeast Asia	Viet Nam	Subnational	International Centre for Eye Health (ICEH). Vietnam - Binh Duong Rapid Assessment of Avoidable Blindness Survey 2015. Grootebroek, Netherlands: RAAB Repository.
Southeast Asia, East Asia, and Oceania	Southeast Asia	Viet Nam	Subnational	International Centre for Eye Health (ICEH). Vietnam - Binh Phuoc Rapid Assessment of Avoidable Blindness 2007. Grootebroek, Netherlands: RAAB Repository.
Southeast Asia, East Asia, and Oceania	Southeast Asia	Viet Nam	Subnational	International Centre for Eye Health (ICEH). Vietnam - Binh Phuoc Rapid Assessment of Cataract Surgical Services 2002. Grootebroek, Netherlands: RAAB Repository.

Southeast Asia, East Asia, and Oceania	Southeast Asia	Viet Nam	Subnational	International Centre for Eye Health (ICEH). Vietnam - Ca Mau Rapid Assessment of Avoidable Blindness Survey 2015. Grootebroek, Netherlands: RAAB Repository.
Southeast Asia, East Asia, and Oceania	Southeast Asia	Viet Nam	Subnational	International Centre for Eye Health (ICEH). Vietnam - Can Tho Rapid Assessment of Avoidable Blindness 2007. Grootebroek, Netherlands: RAAB Repository.
Southeast Asia, East Asia, and Oceania	Southeast Asia	Viet Nam	Subnational	International Centre for Eye Health (ICEH). Vietnam - Dien Bien Rapid Assessment of Avoidable Blindness Survey 2015. Grootebroek, Netherlands: RAAB Repository.
Southeast Asia, East Asia, and Oceania	Southeast Asia	Viet Nam	Subnational	International Centre for Eye Health (ICEH). Vietnam - Gia Lai Rapid Assessment of Avoidable Blindness 2007. Grootebroek, Netherlands: RAAB Repository.
Southeast Asia, East Asia, and Oceania	Southeast Asia	Viet Nam	Subnational	International Centre for Eye Health (ICEH). Vietnam - Gia Lai Rapid Assessment of Avoidable Blindness Survey 2015. Grootebroek, Netherlands: RAAB Repository.
Southeast Asia, East Asia, and Oceania	Southeast Asia	Viet Nam	Subnational	International Centre for Eye Health (ICEH). Vietnam - Gia Lai Rapid Assessment of Cataract Surgical Services 2002. Grootebroek, Netherlands: RAAB Repository.
Southeast Asia, East Asia, and Oceania	Southeast Asia	Viet Nam	Subnational	International Centre for Eye Health (ICEH). Vietnam - Ha Tay Rapid Assessment of Avoidable Blindness 2007. Grootebroek, Netherlands: RAAB Repository.
Southeast Asia, East Asia, and Oceania	Southeast Asia	Viet Nam	Subnational	International Centre for Eye Health (ICEH). Vietnam - Ha Tay Rapid Assessment of Cataract Surgical Services 2000. Grootebroek, Netherlands: RAAB Repository.
Southeast Asia, East Asia, and Oceania	Southeast Asia	Viet Nam	Subnational	International Centre for Eye Health (ICEH). Vietnam - Hai Phong Rapid Assessment of Avoidable Blindness 2007. Grootebroek, Netherlands: RAAB Repository.
Southeast Asia, East Asia, and Oceania	Southeast Asia	Viet Nam	Subnational	International Centre for Eye Health (ICEH). Vietnam - Hai Phong Rapid Assessment of Cataract Surgical Services 2000. Grootebroek, Netherlands: RAAB Repository.
Southeast Asia, East Asia, and Oceania	Southeast Asia	Viet Nam	Subnational	International Centre for Eye Health (ICEH). Vietnam - Ho Chi Minh Rapid Assessment of Avoidable Blindness 2007. Grootebroek, Netherlands: RAAB Repository.
Southeast Asia, East Asia, and Oceania	Southeast Asia	Viet Nam	Subnational	International Centre for Eye Health (ICEH). Vietnam - Hue Rapid Assessment of Avoidable Blindness 2007. Grootebroek, Netherlands: RAAB Repository.

Southeast Asia, East Asia, and Oceania	Southeast Asia	Viet Nam	Subnational	International Centre for Eye Health (ICEH). Vietnam - Lam Dong Rapid Assessment of Avoidable Blindness Survey 2015. Grootebroek, Netherlands: RAAB Repository.
Southeast Asia, East Asia, and Oceania	Southeast Asia	Viet Nam	Subnational	International Centre for Eye Health (ICEH). Vietnam - Lao Cai Rapid Assessment of Avoidable Blindness 2007. Grootebroek, Netherlands: RAAB Repository.
Southeast Asia, East Asia, and Oceania	Southeast Asia	Viet Nam	Subnational	International Centre for Eye Health (ICEH). Vietnam - Nam Dinh Rapid Assessment of Avoidable Blindness Survey 2015. Grootebroek, Netherlands: RAAB Repository.
Southeast Asia, East Asia, and Oceania	Southeast Asia	Viet Nam	Subnational	International Centre for Eye Health (ICEH). Vietnam - Nghe An Rapid Assessment of Avoidable Blindness 2007. Grootebroek, Netherlands: RAAB Repository.
Southeast Asia, East Asia, and Oceania	Southeast Asia	Viet Nam	Subnational	International Centre for Eye Health (ICEH). Vietnam - Nghe An Rapid Assessment of Avoidable Blindness 2012. Grootebroek, Netherlands: RAAB Repository.
Southeast Asia, East Asia, and Oceania	Southeast Asia	Viet Nam	Subnational	International Centre for Eye Health (ICEH). Vietnam - Nghe An Rapid Assessment of Cataract Surgical Services 2000. Grootebroek, Netherlands: RAAB Repository.
Southeast Asia, East Asia, and Oceania	Southeast Asia	Viet Nam	Subnational	International Centre for Eye Health (ICEH). Vietnam - Ninh Thuan Rapid Assessment of Avoidable Blindness 2007. Grootebroek, Netherlands: RAAB Repository.
Southeast Asia, East Asia, and Oceania	Southeast Asia	Viet Nam	Subnational	International Centre for Eye Health (ICEH). Vietnam - Phu Tho Rapid Assessment of Avoidable Blindness 2007. Grootebroek, Netherlands: RAAB Repository.
Southeast Asia, East Asia, and Oceania	Southeast Asia	Viet Nam	Subnational	International Centre for Eye Health (ICEH). Vietnam - Phu Tho Rapid Assessment of Avoidable Blindness Survey 2015. Grootebroek, Netherlands: RAAB Repository.
Southeast Asia, East Asia, and Oceania	Southeast Asia	Viet Nam	Subnational	International Centre for Eye Health (ICEH). Vietnam - Phu Tho Rapid Assessment of Cataract Surgical Services 2000. Grootebroek, Netherlands: RAAB Repository.
Southeast Asia, East Asia, and Oceania	Southeast Asia	Viet Nam	Subnational	International Centre for Eye Health (ICEH). Vietnam - Quang Nam Rapid Assessment of Avoidable Blindness 2012. Grootebroek, Netherlands: RAAB Repository.
Southeast Asia, East Asia, and Oceania	Southeast Asia	Viet Nam	Subnational	International Centre for Eye Health (ICEH). Vietnam - Quang Ngai Rapid Assessment of Avoidable Blindness

				Survey 2015. Grootebroek, Netherlands: RAAB Repository.
Southeast Asia, East Asia, and Oceania	Southeast Asia	Viet Nam	Subnational	International Centre for Eye Health (ICEH). Vietnam - Quang Tri Rapid Assessment of Avoidable Blindness Survey 2015. Grootebroek, Netherlands: RAAB Repository.
Southeast Asia, East Asia, and Oceania	Southeast Asia	Viet Nam	Subnational	International Centre for Eye Health (ICEH). Vietnam - Son La Rapid Assessment of Avoidable Blindness 2010. Grootebroek, Netherlands: RAAB Repository.
Southeast Asia, East Asia, and Oceania	Southeast Asia	Viet Nam	Subnational	International Centre for Eye Health (ICEH). Vietnam - Thai Nguyen Rapid Assessment of Avoidable Blindness 2007. Grootebroek, Netherlands: RAAB Repository.
Southeast Asia, East Asia, and Oceania	Southeast Asia	Viet Nam	Subnational	International Centre for Eye Health (ICEH). Vietnam - Thanh Hoa Rapid Assessment of Avoidable Blindness 2011. Grootebroek, Netherlands: RAAB Repository.
Southeast Asia, East Asia, and Oceania	Southeast Asia	Viet Nam	Subnational	International Centre for Eye Health (ICEH). Vietnam - Tien Giang Rapid Assessment of Avoidable Blindness 2007. Grootebroek, Netherlands: RAAB Repository.
Southeast Asia, East Asia, and Oceania	Southeast Asia	Viet Nam	Subnational	International Centre for Eye Health (ICEH). Vietnam - Tien Giang Rapid Assessment of Avoidable Blindness Survey 2015. Grootebroek, Netherlands: RAAB Repository.
Southeast Asia, East Asia, and Oceania	Southeast Asia	Viet Nam	Subnational	International Centre for Eye Health (ICEH). Vietnam - Tien Giang Rapid Assessment of Cataract Surgical Services 2000. Grootebroek, Netherlands: RAAB Repository.
Southeast Asia, East Asia, and Oceania	Southeast Asia	Viet Nam	Subnational	International Centre for Eye Health (ICEH). Vietnam - Tuyen Quang Rapid Assessment of Avoidable Blindness Survey 2015. Grootebroek, Netherlands: RAAB Repository.
Southeast Asia, East Asia, and Oceania	Southeast Asia	Viet Nam	Subnational	International Centre for Eye Health (ICEH). Vietnam - Vung Tau Rapid Assessment of Avoidable Blindness Survey 2015. Grootebroek, Netherlands: RAAB Repository.
Sub-Saharan Africa	Central Sub-Saharan Africa	Democratic Republic of the Congo	Subnational	International Centre for Eye Health (ICEH). Democratic Republic of Congo - Orientale Rapid Assessment of Avoidable Blindness 2015. Grootebroek, Netherlands: RAAB Repository.

Sub-Saharan Africa	Eastern Sub-Saharan Africa	Burundi	Subnational	International Centre for Eye Health (ICEH). Burundi - Ngozi and Kayanza Rapid Assessment of Avoidable Blindness 2010. Grootebroek, Netherlands: RAAB Repository.
Sub-Saharan Africa	Eastern Sub-Saharan Africa	Eritrea	National	International Centre for Eye Health (ICEH), Ministry of Health (Eritrea). Eritrea Rapid Assessment for Avoidable Blindness Survey 2008.
Sub-Saharan Africa	Eastern Sub-Saharan Africa	Ethiopia	National	Berhane Y, Worku A, Bejiga A, Adamu L, Alemayehu W, Bedri A, Haile Z, Ayalew A, Adamu Y, Gebre T, Kebede TD, West E, West S. Prevalence and causes of blindness and Low Vision in Ethiopia. Ethiop J Health Dev. 2008; 21(3): 204-10.
Sub-Saharan Africa	Eastern Sub-Saharan Africa	Kenya	National	Schwab L, Steinkuller PG. Visual disability and blindness secondary to refractive errors in Africa. Soc Sci Med. 1983; 17(22): 1751-4.
Sub-Saharan Africa	Eastern Sub-Saharan Africa	Kenya	National	Whitfield R, Schwab L, Ross-Degnan D, Steinkuller P, Swartwood J. Blindness and eye disease in Kenya: ocular status survey results from the Kenya Rural Blindness Prevention Project. Br J Ophthalmol. 1990; 74(6): 333-40.
Sub-Saharan Africa	Eastern Sub-Saharan Africa	Kenya	Subnational	International Centre for Eye Health (ICEH). Kenya - Embu Rapid Assessment of Avoidable Blindness 2007. Grootebroek, Netherlands: RAAB Repository.
Sub-Saharan Africa	Eastern Sub-Saharan Africa	Kenya	Subnational	International Centre for Eye Health (ICEH). Kenya - Kericho Rapid Assessment of Avoidable Blindness 2007. Grootebroek, Netherlands: RAAB Repository.
Sub-Saharan Africa	Eastern Sub-Saharan Africa	Kenya	Subnational	International Centre for Eye Health (ICEH). Kenya - Kwale Rapid Assessment of Avoidable Blindness 2011. Grootebroek, Netherlands: RAAB Repository.
Sub-Saharan Africa	Eastern Sub-Saharan Africa	Kenya	Subnational	International Centre for Eye Health (ICEH). Kenya - Nakuru Rapid Assessment of Avoidable Blindness 2005. Grootebroek, Netherlands: RAAB Repository.
Sub-Saharan Africa	Eastern Sub-Saharan Africa	Madagascar	Subnational	International Centre for Eye Health (ICEH). Madagascar - Atsinanana Rapid Assessment of Avoidable Blindness 2011. Grootebroek, Netherlands: RAAB Repository.
Sub-Saharan Africa	Eastern Sub-Saharan Africa	Malawi	Subnational	International Centre for Eye Health (ICEH). Malawi - Southern Region Rapid Assessment of Avoidable

				Blindness 2009-2010. Grootebroek, Netherlands: RAAB Repository.
Sub-Saharan Africa	Eastern Sub-Saharan Africa	Mozambique	Subnational	International Centre for Eye Health (ICEH). Mozambique - Nampula Rapid Assessment of Avoidable Blindness Survey 2011. Grootebroek, Netherlands: RAAB Repository, 2014.
Sub-Saharan Africa	Eastern Sub-Saharan Africa	Mozambique	Subnational	International Centre for Eye Health (ICEH). Mozambique - Sofala Division Rapid Assessment of Avoidable Blindness 2012. Grootebroek, Netherlands: RAAB Repository.
Sub-Saharan Africa	Eastern Sub-Saharan Africa	Rwanda	National	International Centre for Eye Health (ICEH). Rwanda Rapid Assessment of Avoidable Blindness Survey 2015. Grootebroek, Netherlands: RAAB Repository, 2014.
Sub-Saharan Africa	Eastern Sub-Saharan Africa	Rwanda	Subnational	Mathenge W NJ, Limburg H, Kuper H. Rapid assessment of avoidable blindness in Western Rwanda: blindness in a postconflict setting. PLoS Med. 2007; 4(7).
Sub-Saharan Africa	Eastern Sub-Saharan Africa	Uganda	Subnational	International Centre for Eye Health (ICEH). Uganda - Hoima Rapid Assessment of Avoidable Blindness 2013. Grootebroek, Netherlands: RAAB Repository.
Sub-Saharan Africa	Eastern Sub-Saharan Africa	Uganda	Subnational	International Centre for Eye Health (ICEH). Uganda - Karamoja Rapid Assessment of Avoidable Blindness Survey 2015. Grootebroek, Netherlands: RAAB Repository.[Forthcoming].
Sub-Saharan Africa	Eastern Sub-Saharan Africa	Uganda	Subnational	International Centre for Eye Health (ICEH). Uganda - Mubende Rapid Assessment of Avoidable Blindness 2012. Grootebroek, Netherlands: RAAB Repository.
Sub-Saharan Africa	Eastern Sub-Saharan Africa	Uganda	Subnational	International Centre for Eye Health (ICEH). Uganda - Western Ntungamo Rapid Assessment of Avoidable Blindness Survey 2011. Grootebroek, Netherlands: RAAB Repository. [Forthcoming}.
Sub-Saharan Africa	Eastern Sub-Saharan Africa	Uganda	Subnational	Mbulaiteye SM, Reeves BC, Karabalinde A, Ruberantwari A, Mulwanyi F, Whitworth JAG, Johnson GJ. Evaluation of E-optotypes as a screening test and the prevalence and causes of visual loss in a rural population in SW Uganda. Ophthalmic Epidemiol. 2002; 9(4): 251-62.
Sub-Saharan Africa	Eastern Sub-Saharan Africa	United Republic of Tanzania	Subnational	International Centre for Eye Health (ICEH). Tanzania - Kigoma Rapid Assessment of Avoidable Blindness Survey

				2011. Grootebroek, Netherlands: RAAB Repository, 2010. [Unpublished].
Sub-Saharan Africa	Eastern Sub-Saharan Africa	United Republic of Tanzania	Subnational	International Centre for Eye Health (ICEH). Tanzania - Kilimanjaro Rapid Assessment of Avoidable Blindness 2007. Grootebroek, Netherlands: RAAB Repository.
Sub-Saharan Africa	Eastern Sub-Saharan Africa	United Republic of Tanzania	Subnational	International Centre for Eye Health (ICEH). Tanzania - Singida Rapid Assessment of Avoidable Blindness Survey 2017. Grootebroek, Netherlands: RAAB Repository.
Sub-Saharan Africa	Eastern Sub-Saharan Africa	United Republic of Tanzania	Subnational	International Centre for Eye Health (ICEH). Tanzania - Zanzibar Rapid Assessment of Avoidable Blindness 2007.
Sub-Saharan Africa	Eastern Sub-Saharan Africa	United Republic of Tanzania	Subnational	Rapoza PA, West SK, Katala SJ, Taylor HR. Prevalence and causes of vision loss in central Tanzania. Int J Ophthalmol. 1991; 15(2): 123-9.
Sub-Saharan Africa	Eastern Sub-Saharan Africa	Zambia	Subnational	Lindfield R, Griffiths U, Bozzani F, Mumba M, Munsanje J. A Rapid Assessment of Avoidable Blindness in Southern Zambia. PLoS One. 2012; 7(6): e38483.
Sub-Saharan Africa	Southern Sub-Saharan Africa	Botswana	National	International Centre for Eye Health (ICEH). Botswana Rapid Assessment of Avoidable Blindness 2013-2014. Grootebroek, Netherlands: RAAB Repository.
Sub-Saharan Africa	Southern Sub-Saharan Africa	South Africa	Subnational	Cockburn N, Steven D, Lecuona K, Joubert F, Rogers G, Cook C, Polack S. Prevalence, Causes and Socio-Economic Determinants of Vision Loss in Cape Town, South Africa. Atashili J, editor. PLoS One. 2012; 7(2): e30718.
Sub-Saharan Africa	Southern Sub-Saharan Africa	South Africa	Subnational	International Centre for Eye Health (ICEH). South Africa Rapid Assessment of Avoidable Blindness Survey 2010. Grootebroek, Netherlands: RAAB Repository, 2014.
Sub-Saharan Africa	Southern Sub-Saharan Africa	South Africa	Subnational	Salmon JF, Mermoud A, Ivey A, Swanevelder SA, Hoffman M. The prevalence of primary angle closure glaucoma and open angle glaucoma in Mamre, western Cape, South Africa. Arch Ophthalmol. 1993; 111(9): 1263-9.
Sub-Saharan Africa	Southern Sub-Saharan Africa	Zimbabwe	Subnational	International Centre for Eye Health (ICEH). Zimbabwe - Manicaland Rapid Assessment of Avoidable Blindness Survey 2016. Grootebroek, Netherlands: RAAB Repository.

Sub-Saharan Africa	Western Sub-Saharan Africa	Burkina Faso	Subnational	International Centre for Eye Health (ICEH). Burkina Faso - West Central Rapid Assessment of Avoidable Blindness 2011. Grootebroek, Netherlands: RAAB Repository.
Sub-Saharan Africa	Western Sub-Saharan Africa	Cabo Verde	National	Schémann JF, Inocencio F, de Lourdes Monteiro M, Andrade J, Auzemery A, Guelfi Y. Blindness and low vision in Cape Verde Islands: results of a national eye survey. *Ophthalmic Epidemiol*. 2006; 13(4): 219-26.
Sub-Saharan Africa	Western Sub-Saharan Africa	Gambia	National	International Centre for Eye Health (ICEH). Gambia Rapid Assessment of Avoidable Blindness 2007.
Sub-Saharan Africa	Western Sub-Saharan Africa	Guinea-Bissau	National	International Centre for Eye Health (ICEH). Guinea-Bissau Rapid Assessment of Avoidable Blindness 2010. Grootebroek, Netherlands: RAAB Repository.
Sub-Saharan Africa	Western Sub-Saharan Africa	Mali	Subnational	International Centre for Eye Health (ICEH). Mali - Koulikoro Rapid Assessment of Avoidable Blindness 2008. Grootebroek, Netherlands: RAAB Repository.
Sub-Saharan Africa	Western Sub-Saharan Africa	Mali	Subnational	International Centre for Eye Health (ICEH). Mali - Koulikoro Rapid Assessment of Avoidable Blindness 2011. Grootebroek, Netherlands: RAAB Repository.
Sub-Saharan Africa	Western Sub-Saharan Africa	Mali	Subnational	Kortlang C, Koster JC, Coulibaly S, Dubbeldam RP. Prevalence of blindness and visual impairment in the region of Ségou, Mali. A baseline survey for a primary eye care programme. *Trop Med Int Health*. 1996; 1(3): 314-9.
Sub-Saharan Africa	Western Sub-Saharan Africa	Nigeria	National	Kyari F, Gudlavalleti MVS, Sivsubramaniam S, Gilbert CE, Abdull MM, Entekume G, Foster A. Prevalence of blindness and visual impairment in Nigeria: the National Blindness and Visual Impairment Study. Invest Ophthalmol Vis Sci. 2009; 50(5): 2033-9.
Sub-Saharan Africa	Western Sub-Saharan Africa	Nigeria	Subnational	Adegbehingbe BO, Majengbasan TO. Ocular health status of rural dwellers in south-western Nigeria. Aust J Rural Health. 2007; 15(4): 269-72.
Sub-Saharan Africa	Western Sub-Saharan Africa	Nigeria	Subnational	Adeoti CO. Prevalence and causes of blindness in a tropical African population. West Afr J Med. 2004; 23(3): 249-52.
Sub-Saharan Africa	Western Sub-Saharan Africa	Nigeria	Subnational	Adeoye A. Survey of blindness in rural communities of south-western Nigeria. Trop Med Int Health. 1996; 1(5): 672-6.

Sub-Saharan Africa	Western Sub-Saharan Africa	Nigeria	Subnational	Ezepue UF. Magnitude and causes of blindness and low vision in Anambra State of Nigeria (results of 1992 point prevalence survey). Public Health. 1997; 111(5): 305-9.
Sub-Saharan Africa	Western Sub-Saharan Africa	Nigeria	Subnational	International Centre for Eye Health (ICEH). Nigeria - Sokoto Rapid Assessment of Avoidable Blindness 2016. Grootebroek, Netherlands: RAAB Repository.
Sub-Saharan Africa	Western Sub-Saharan Africa	Nigeria	Subnational	Odugbo OP, Mpyet CD, Chiroma MR, Aboje AO. Cataract blindness, surgical coverage, outcome, and barriers to uptake of cataract services in Plateau State, Nigeria. Middle East Afr J Ophthalmol. 2012; 19(3): 282-8.
Sub-Saharan Africa	Western Sub-Saharan Africa	Nigeria	Subnational	Rabiu MM, Muhammed N. Rapid Assessment of cataract surgical services in Birnin-Kebbi local government area of Kebbi State, Nigeria. Ophthalmic Epidemiol. 2008; 15(6): 359-65.
Sub-Saharan Africa	Western Sub-Saharan Africa	Senegal	Subnational	International Centre for Eye Health (ICEH). Senegal - Fatick Rapid Assessment of Avoidable Blindness 2010. Grootebroek, Netherlands: RAAB Repository.
Sub-Saharan Africa	Western Sub-Saharan Africa	Senegal	Subnational	International Centre for Eye Health (ICEH). Senegal - Kaolack Rapid Assessment of Avoidable Blindness 2010. Grootebroek, Netherlands: RAAB Repository.
Sub-Saharan Africa	Western Sub-Saharan Africa	Sierra Leone	National	International Centre for Eye Health (ICEH). Sierra Leone Rapid Assessment of Avoidable Blindness 2010-2011.
