# Supplementary material for: Global estimates on the number of people blind or visually impaired by age-related macular degeneration: a meta-analysis from 2000 to 2020
Source: Eye (Lond). 2024 Jul 4;38(11):2070–82. doi: 10.1038/s41433-024-03050-z (PMC11269688; doi:10.1038/s41433-024-03050-z)
Supplement: Supplementary file 4 — Supplementary material [file 41433_2024_3050_MOESM4_ESM.docx]

**Supplementary material**

**Figure 1:** Crude prevalence of blindness due to age-related macular degeneration per age groups and world regions in 2020

**Figure 2:** Crude prevalence of MSVI due to age-related macular degeneration per age groups and world regions in 2020

**Appendix Table 1:** studies that provided age-related macular degeneration prevalence estimates arranged by World GBD Region
