# Supplementary material for: Global estimates on the number of people blind or visually impaired by age-related macular degeneration: a meta-analysis from 2000 to 2020
Source: Eye (Lond). 2024 Jul 4;38(11):2070–82. doi: 10.1038/s41433-024-03050-z (PMC11269688; doi:10.1038/s41433-024-03050-z)
Supplement: Supplementary file 5 — Supplementary Appendix [file 41433_2024_3050_MOESM5_ESM.docx]

**Appendix: Contributions by Authors**

# GBD 2019 Blindness and Vision Impairment Collaborators

### Providing data or critical feedback on data sources

Yohannes Habtegiorgis Abate, Tadele Girum Girum Adal, Kishor Adhikari, Antonella Agodi, Williams Agyemang-Duah, Fares Alahdab, Syed Shujait Shujait Ali, Louay Almidani, Sofia Androudi, Jalal Arabloo, Alessandro Arrigo, Seyyed Shamsadin Athari, Desta Debalkie Atnafu, Alok Atreya, Yared Asmare Aynalem, Zewdu Bishaw Aynalem, Ahmed Y Azzam, Sara Bagherieh, Martina Barchitta, Mainak Bardhan, Till Winfried Bärnighausen, Nebiyou Simegnew Bayileyegn, Ahmet Begde, Babak Behnam, Akshaya Srikanth Bhagavathula, Sonu Bhaskar, Gurjit Kaur Bhatti, Jasvinder Singh Bhatti, Bagas Suryo Bintoro, Rupert Bourne, Tasanee Braithwaite, Paul Svitil Briant, Florentino Luciano Caetano dos Santos, Muthia Cenderadewi, Vijay Kumar Chattu, Dinh-Toi Chu, Maria Vittoria Cicinelli, Natália Cruz-Martins, Xiaochen Dai, Maedeh Dastmardi, Nikolaos Dervenis, Vinoth Gnana Chellaiyan Devanbu, Joshua R Ehrlich, Michael Ekholuenetale, Temitope Cyrus Ekundayo, Iman El Sayed, Ambaw Abebaw Emrie, Adeniyi Francis Fagbamigbe, Hossein Farrokhpour, Ali Fatehizadeh, Alireza Feizkhah, Arthur G Fernandes, Lorenzo Ferro Desideri, Seth Flaxman, Kayode Raphael Fowobaje, João M Furtado, Tilaye Gebru Gebi, Brhane Gebremariam, Molalegn Mesele Gesese, Fariba Ghassemi, Sherief Ghozy, Mahaveer Golechha, Pouya Goleij, Sapna Gupta, Veer Bala Gupta, Vivek Kumar Gupta, Teklehaimanot Gereziher Haile, Arvin Haj-Mirzaian, Sung Hwi Hong, Praveen Hoogar, Mehdi Hosseinzadeh, Chengxi Hu, Hong-Han Huynh, Chidozie C D Iwu, Mihajlo Jakovljevic, Shubha Jayaram, Jost B Jonas, Charity Ehimwenma Joshua, Gebisa Guyasa Kabito, Laleh R Kalankesh, Himal Kandel, Gbenga A Kayode, Shemsu Kedir, Yousef Saleh Khader, Himanshu Khajuria, Mahalaqua Nazli Khatib, Yun Jin Kim, Adnan Kisa, Sezer Kisa, Soewarta Kosen, Kewal Krishan, Chandrakant Lahariya, Tri Laksono, Dharmesh Kumar Lal, Trang Diep Thanh Le, Munjae Lee, Seung Won Lee, Nicolas Leveziel, Stephen S Lim, Xuefeng Liu, Razzagh Mahmoudi, Kashish Malhotra, Roy Rillera Marzo, Andrea Maugeri, Tesfahun Mekene Meto, Soheil Mohammadi, Ali H Mokdad, Mohammad Ali Moni, Maryam Moradi, Admir Mulita, Kovin S Naidoo, Ganesh R Naik, Shumaila Nargus, Zuhair S Natto, Biswa Prakash Nayak, Dang H Nguyen, Hien Quang Nguyen, Phat Tuan Nguyen, Van Thanh Nguyen, Robina Khan Niazi, Ogochukwu Janet Nzoputam, Ismail A Odetokun, Andrew T Olagunju, Matthew Idowu Olatubi, Obinna E Onwujekwe, Uchechukwu Levi Osuagwu, Mayowa O Owolabi, Jagadish Rao Padubidri, Jay Patel, Shrikant Pawar, Arokiasamy Perianayagam, Hoang Tran Pham, Fakher Rahim, Vafa Rahimi-Movaghar, Ahmed Mustafa Rashid, Annisa Utami Rauf, Elrashdy Moustafa Mohamed Redwan, Serge Resnikoff, Zahra Saadatian, Siamak Sabour, Basema Saddik, Umar Saeed, Sare Safi, Sher Zaman Safi, Narjes Saheb Sharif-Askari, Joseph W Sakshaug, Vijaya Paul Samuel, Abdallah M Samy, Monika Sawhney, Mete Saylan, Sayed Mansoor Sediqi, Yashendra Sethi, Allen Seylani, Jaffer Shah, Masood Ali Shaikh, Muhammad Aaqib Shamim, Maryam Shayan, Aminu Shittu, Jasvinder A Singh, Paramdeep Singh, Chandrashekhar T Sreeramareddy, Jaimie D Steinmetz, Ian Tapply, Hugh R Taylor, Guesh Mebrahtom Tsegay, Saif Ullah, Muhammad Umair, Sahel Valadan Tahbaz, Theo Vos, Dong Keon Yon, Naohiro Yonemoto, Mikhail Sergeevich Zastrozhin, and Magdalena Zielińska.

### Developing methods or computational machinery

Alessandro Arrigo, Desta Debalkie Atnafu, Ahmed Y Azzam, Akshaya Srikanth Bhagavathula, Rupert Bourne, Paul Svitil Briant, Kaleb Coberly, Xiaochen Dai, Maedeh Dastmardi, Michael Ekholuenetale, Mehdi Emamverdi, Ayesha Fahim, Ali Fatehizadeh, Arthur G Fernandes, Seth Flaxman, Sherief Ghozy, Mehdi Hosseinzadeh, Hong-Han Huynh, Jost B Jonas, Charity Ehimwenma Joshua, Mahalaqua Nazli Khatib, Adnan Kisa, Chandrakant Lahariya, Nicolas Leveziel, Razzagh Mahmoudi, Ali H Mokdad, Mohammad Ali Moni, Admir Mulita, Hien Quang Nguyen, Phat Tuan Nguyen, Van Thanh Nguyen, Michal Ordak, Jagadish Rao Padubidri, Hoang Tran Pham, Premkumar Ramasubramani, Umar Saeed, Abdallah M Samy, Monika Sawhney, Tabassom Sedighi, Jaimie D Steinmetz, Ian Tapply, Hugh R Taylor, Muhammad Umair, Theo Vos, and Peng Zheng.

### Providing critical feedback on methods or results

Yohannes Habtegiorgis Abate, Mohammad Abdollahi, Tadele Girum Girum Adal, Isaac Yeboah Addo, Kishor Adhikari, Antonella Agodi, Aqeel Ahmad, Hamid Ahmadieh, Hooman Ahmadzadeh, Fares Alahdab, Ahmad Samir Alfaar, Robert Kaba Alhassan, Syed Shujait Shujait Ali, Louay Almidani, Sofia Androudi, Anayochukwu Edward Anyasodor, Jalal Arabloo, Alessandro Arrigo, Mubarek Yesse Ashemo, Seyyed Shamsadin Athari, Desta Debalkie Atnafu, Alok Atreya, Melese Kitu Ayalew, Yared Asmare Aynalem, Zewdu Bishaw Aynalem, Ahmed Y Azzam, Sara Bagherieh, Ruhai Bai, Martina Barchitta, Mainak Bardhan, Till Winfried Bärnighausen, Nebiyou Simegnew Bayileyegn, Fatemeh Bazvand, Ahmet Begde, Babak Behnam, Akshaya Srikanth Bhagavathula, Sonu Bhaskar, Gurjit Kaur Bhatti, Jasvinder Singh Bhatti, Bagas Suryo Bintoro, Rupert Bourne, Tasanee Braithwaite, Paul Svitil Briant, Katrin Burkart, Yasser Bustanji, Florentino Luciano Caetano dos Santos, Vera L A Carneiro, Muthia Cenderadewi, Vijay Kumar Chattu, Dinh-Toi Chu, Natália Cruz-Martins, Omid Dadras, Xiaochen Dai, Ana Maria Dascalu, Mohsen Dashti, Anna Dastiridou, Maedeh Dastmardi, Xinlei Deng, Nikolaos Dervenis, Vinoth Gnana Chellaiyan Devanbu, Mengistie Diress, Shirin Djalalinia, Michael Ekholuenetale, Temitope Cyrus Ekundayo, Iman El Sayed, Muhammed Elhadi, Mehdi Emamverdi, Ambaw Abebaw Emrie, Adeniyi Francis Fagbamigbe, Ayesha Fahim, Umar Farooq, Hossein Farrokhpour, Ali Fatehizadeh, Alireza Feizkhah, Arthur G Fernandes, Lorenzo Ferro Desideri, Getahun Fetensa, Bikila Regassa Feyisa, Florian Fischer, Seth Flaxman, Ali Forouhari, Matteo Foschi, Kayode Raphael Fowobaje, João M Furtado, Aravind P Gandhi, Tilaye Gebru Gebi, Miglas W Gebregergis, Mesfin Gebrehiwot, Brhane Gebremariam, Molalegn Mesele Gesese, Khalil Ghasemi Falavarjani, Fariba Ghassemi, Sherief Ghozy, Mahaveer Golechha, Sapna Gupta, Veer Bala Gupta, Vivek Kumar Gupta, Teklehaimanot Gereziher Haile, Semira Goitom Hailu, Arvin Haj-Mirzaian, Aram Halimi, Shahin Hallaj, Billy Randall Hammond, Ikramul Hasan, Hamidreza Hasani, Hossein Hassanian-Moghaddam, Mahsa Heidari-Foroozan, Sung Hwi Hong, Praveen Hoogar, Mehdi Hosseinzadeh, Chengxi Hu, Hong-Han Huynh, Mustapha Immurana, Chidozie C D Iwu, Louis Jacob, Mihajlo Jakovljevic, Shubha Jayaram, Mohammad Jokar, Jost B Jonas, Nitin Joseph, Charity Ehimwenma Joshua, Gebisa Guyasa Kabito, Laleh R Kalankesh, Sagarika Kamath, Himal Kandel, Ibraheem M Karaye, Gbenga A Kayode, Shemsu Kedir, Yousef Saleh Khader, Himanshu Khajuria, Moawiah Mohammad Khatatbeh, Mahalaqua Nazli Khatib, Zahra Khorrami, Yun Jin Kim, Adnan Kisa, Sezer Kisa, Ai Koyanagi, Kewal Krishan, Chandrakant Lahariya, Tri Laksono, Dharmesh Kumar Lal, Van Charles Lansingh, Trang Diep Thanh Le, Munjae Lee, Seung Won Lee, Wei-Chen Lee, Nicolas Leveziel, Stephen S Lim, Xuefeng Liu, Alireza Mahmoudi, Razzagh Mahmoudi, Kashish Malhotra, Vahid Mansouri, Roy Rillera Marzo, Andrea Maugeri, Colm McAlinden, Tesfahun Mekene Meto, Abera M Mersha, Tomislav Mestrovic, Ephrem Tesfaye Mihretie, Mehdi Mirzaei, Prasanna Mithra, Nouh Saad Mohamed, Soheil Mohammadi, Abdulwase Mohammed, Ali H Mokdad, Hossein Molavi Vardanjani, Mohammad Ali Moni, Fateme Montazeri, Maryam Moradi, Ahmed Nuru Muhamed, Admir Mulita, Ganesh R Naik, Shumaila Nargus, Zuhair S Natto, Biswa Prakash Nayak, Mohammad Negaresh, Hadush Negash, Seyed Aria Nejadghaderi, Dang H Nguyen, Hien Quang Nguyen, Phat Tuan Nguyen, Van Thanh Nguyen, Robina Khan Niazi, Ogochukwu Janet Nzoputam, Ismail A Odetokun, Andrew T Olagunju, Matthew Idowu Olatubi, Obinna E Onwujekwe, Michal Ordak, Uchechukwu Levi Osuagwu, Nikita Otstavnov, Mayowa O Owolabi, Jagadish Rao Padubidri, Parsa Panahi, Ashok Pandey, Shahina Pardhan, Jay Patel, Shrikant Pawar, Arokiasamy Perianayagam, Ionela-Roxana Petcu, Hoang Tran Pham, Ibrahim Qattea, Pankaja Raghav Raghav, Fakher Rahim, Vafa Rahimi-Movaghar, Mosiur Rahman, Premkumar Ramasubramani, Ahmed Mustafa Rashid, Annisa Utami Rauf, Elrashdy Moustafa Mohamed Redwan, Serge Resnikoff, Nazila Rezaei, Priyanka Roy, Zahra Saadatian, Siamak Sabour, Basema Saddik, Umar Saeed, Sare Safi, Sher Zaman Safi, Narjes Saheb Sharif-Askari, Joseph W Sakshaug, Mohamed A Saleh, Yoseph Leonardo Samodra, Vijaya Paul Samuel, Abdallah M Samy, Monika Sawhney, Mete Saylan, Sayed Mansoor Sediqi, Yashendra Sethi, Jaffer Shah, Samiah Shahid, Masood Ali Shaikh, Muhammad Aaqib Shamim, Maryam Shayan, Mika Shigematsu, Aminu Shittu, Seyed Afshin Shorofi, Emmanuel Edwar Siddig, Juan Carlos Silva, Jasvinder A Singh, Paramdeep Singh, Eirini Skiadaresi, Chandrashekhar T Sreeramareddy, Vladimir I Starodubov, Ian Tapply, Birhan Tsegaw Taye, Jansje Henny Vera Ticoalu, Guesh Mebrahtom Tsegay, Saif Ullah, Muhammad Umair, Sahel Valadan Tahbaz, Theo Vos, Nuwan Darshana Wickramasinghe, Guadie Sharew Wondimagegn, Arzu Yiğit, Dong Keon Yon, Naohiro Yonemoto, Yuyi You, Mikhail Sergeevich Zastrozhin, Hanqing Zhao, and Magdalena Zielińska.

### Drafting the work or revising it critically for important intellectual content

Yohannes Habtegiorgis Abate, Mohammad Abdollahi, Tadele Girum Girum Adal, Isaac Yeboah Addo, Prerna Agarwal, Antonella Agodi, Hamid Ahmadieh, Hooman Ahmadzadeh, Fares Alahdab, Ahmad Samir Alfaar, Robert Kaba Alhassan, Syed Shujait Shujait Ali, Louay Almidani, Abhishek Anil, Anayochukwu Edward Anyasodor, Jalal Arabloo, Alessandro Arrigo, Seyyed Shamsadin Athari, Desta Debalkie Atnafu, Alok Atreya, Melese Kitu Ayalew, Ahmed Y Azzam, Sara Bagherieh, Martina Barchitta, Mainak Bardhan, Till Winfried Bärnighausen, Maurizio Battaglia Parodi, Ahmet Begde, Akshaya Srikanth Bhagavathula, Sonu Bhaskar, Gurjit Kaur Bhatti, Jasvinder Singh Bhatti, Marina G Birck, Rupert Bourne, Tasanee Braithwaite, Paul Svitil Briant, Yasser Bustanji, Florentino Luciano Caetano dos Santos, Muthia Cenderadewi, Vijay Kumar Chattu, Dinh-Toi Chu, Maria Vittoria Cicinelli, Natália Cruz-Martins, Ana Maria Dascalu, Anna Dastiridou, Maedeh Dastmardi, Nikolaos Dervenis, Joshua R Ehrlich, Michael Ekholuenetale, Iman El Sayed, Muhammed Elhadi, Mehdi Emamverdi, Ambaw Abebaw Emrie, Adeniyi Francis Fagbamigbe, Ayesha Fahim, Ali Fatehizadeh, Arthur G Fernandes, Getahun Fetensa, Florian Fischer, Seth Flaxman, Ali Forouhari, Matteo Foschi, João M Furtado, Aravind P Gandhi, Tilaye Gebru Gebi, Miglas W Gebregergis, Mesfin Gebrehiwot, Brhane Gebremariam, Gebreamlak Gebremedhn Gebremeskel, Yibeltal Yismaw Gela, Fariba Ghassemi, Sherief Ghozy, Sapna Gupta, Veer Bala Gupta, Vivek Kumar Gupta, Teklehaimanot Gereziher Haile, Aram Halimi, Shahin Hallaj, Billy Randall Hammond, Ikramul Hasan, Hamidreza Hasani, Hong-Han Huynh, Mustapha Immurana, Chidozie C D Iwu, Louis Jacob, Abdollah Jafarzadeh, Mihajlo Jakovljevic, Shubha Jayaram, Jost B Jonas, Nitin Joseph, Charity Ehimwenma Joshua, Gebisa Guyasa Kabito, Sagarika Kamath, Himal Kandel, Hengameh Kasraei, Gbenga A Kayode, Shemsu Kedir, Yousef Saleh Khader, Himanshu Khajuria, Moawiah Mohammad Khatatbeh, Mahalaqua Nazli Khatib, Adnan Kisa, Sezer Kisa, Ai Koyanagi, Kewal Krishan, Chandrakant Lahariya, Van Charles Lansingh, Janet L Leasher, Nicolas Leveziel, Xuefeng Liu, Razzagh Mahmoudi, Vahid Mansouri, Roy Rillera Marzo, Andrea Maugeri, Colm McAlinden, Tesfahun Mekene Meto, Tomislav Mestrovic, Ephrem Tesfaye Mihretie, Mehdi Mirzaei, Prasanna Mithra, Nouh Saad Mohamed, Soheil Mohammadi, Abdulwase Mohammed, Ali H Mokdad, Hossein Molavi Vardanjani, Mohammad Ali Moni, Fateme Montazeri, Maryam Moradi, Parsa Mousavi, Kovin S Naidoo, Shumaila Nargus, Zuhair S Natto, Biswa Prakash Nayak, Mohammad Negaresh, Hadush Negash, Seyed Aria Nejadghaderi, Dang H Nguyen, Hien Quang Nguyen, Phat Tuan Nguyen, Van Thanh Nguyen, Robina Khan Niazi, Mamoona Noreen, Ogochukwu Janet Nzoputam, Ismail A Odetokun, Andrew T Olagunju, Matthew Idowu Olatubi, Obinna E Onwujekwe, Michal Ordak, Uchechukwu Levi Osuagwu, Nikita Otstavnov, Mayowa O Owolabi, Jagadish Rao Padubidri, Shahina Pardhan, Jay Patel, Venkata Suresh Patthipati, Shrikant Pawar, Arokiasamy Perianayagam, Ionela-Roxana Petcu, Hoang Tran Pham, Ibrahim Qattea, Pankaja Raghav Raghav, Fakher Rahim, Vafa Rahimi-Movaghar, Mohammad Hifz Ur Rahman, Premkumar Ramasubramani, Ahmed Mustafa Rashid, Elrashdy Moustafa Mohamed Redwan, Serge Resnikoff, Nazila Rezaei, Priyanka Roy, Siamak Sabour, Basema Saddik, Umar Saeed, Amene Saghazadeh, Fatemeh Saheb Sharif-Askari, Amirhossein Sahebkar, Saina Salahi, Sarvenaz Salahi, Vijaya Paul Samuel, Abdallah M Samy, Aswini Saravanan, Monika Sawhney, Mete Saylan, Siddharthan Selvaraj, Yashendra Sethi, Allen Seylani, Jaffer Shah, Samiah Shahid, Moyad Jamal Shahwan, Muhammad Aaqib Shamim, Mika Shigematsu, Aminu Shittu, Seyed Afshin Shorofi, Emmanuel Edwar Siddig, Jasvinder A Singh, Paramdeep Singh, Eirini Skiadaresi, Raúl A R C Sousa, Chandrashekhar T Sreeramareddy, Vladimir I Starodubov, Birhan Tsegaw Taye, Miltiadis K Tsilimbaris, Saif Ullah, Muhammad Umair, Sahel Valadan Tahbaz, Theo Vos, Nuwan Darshana Wickramasinghe, Guadie Sharew Wondimagegn, Lin Yang, Arzu Yiğit, Dong Keon Yon, Naohiro Yonemoto, Mikhail Sergeevich Zastrozhin, Makan Ziafati, and Magdalena Zielińska.

### Managing the estimation or publications process

Rupert Bourne, João M Furtado, Ali H Mokdad, and Theo Vos

Vision Loss Expert Group of the Global Burden of Disease Study

### Providing data or critical feedback on data sources

Alessandro Arrigo, Maurizio Battaglia Parodi, Mukharram M Bikbov, Rupert R A Bourne, Tasanee Braithwaite, Alain Bron, Ching-Yu Cheng, Maria Vittoria Cicinelli, Nathan Congdon, Monte A Del Monte, Joshua R Ehrlich, Arthur Fernandes, Seth Flaxman, Tim Fricke, David Friedman, João M Furtado, Gus Gazzard, M Elizabeth Hartnett, Jost B Jonas, Rim Kahloun, John H Kempen, Moncef Khairallah, Rohit C Khanna, Judy E Kim, Van Charles Lansingh, Janet Leasher, Nicolas Leveziel, Kovin S Naidoo, Vinay Nangia, Michal Nowak, Konrad Pesudovs, Tunde Peto, Pradeep Ramulu, Serge Resnikoff, Tabassom Sedighi, Ian Tapply, Hugh Taylor, Fotis Topouzis, Miltiadis Tsilimbaris, Ya Xing Wang, Ningli Wang

### Developing methods or computational machinery

Rupert R A Bourne, Jost B Jonas, Ian Tapply

### Providing critical feedback on methods or results

Alessandro Arrigo, Mukharram M Bikbov, Rupert R A Bourne, Tasanee Braithwaite, Monte A Del Monte, David Friedman, João M Furtado, M Elizabeth Hartnett, Jost B Jonas, Rim Kahloun, John H Kempen, Konrad Pesudovs, Serge Resnikoff, Ian Tapply, Ningli Wang

### Drafting the work or revising it critically for important intellectual content

Alessandro Arrigo, Mukharram M Bikbov, Rupert R A Bourne, Tasanee Braithwaite, Nathan Congdon, Monte A Del Monte, João M Furtado, M Elizabeth Hartnett, Jost B Jonas, Janet Leasher, Konrad Pesudovs

### Managing the estimation or publications process

Rupert R A Bourne, João M Furtado, Jost B Jonas
